# Connectomic Insights into Topologically Centralized Network Edges and Relevant Motifs in the Human Brain

**DOI:** 10.3389/fnhum.2016.00158

**Published:** 2016-04-19

**Authors:** Mingrui Xia, Qixiang Lin, Yanchao Bi, Yong He

**Affiliations:** State Key Laboratory of Cognitive Neuroscience and Learning and IDG/McGovern Institute for Brain Research, Beijing Normal UniversityBeijing, China

**Keywords:** connectomics, diffusion MRI, hub, rich-club, motif, microstructure

## Abstract

White matter (WM) tracts serve as important material substrates for information transfer across brain regions. However, the topological roles of WM tracts in global brain communications and their underlying microstructural basis remain poorly understood. Here, we employed diffusion magnetic resonance imaging and graph-theoretical approaches to identify the pivotal WM connections in human whole-brain networks and further investigated their wiring substrates (including WM microstructural organization and physical consumption) and topological contributions to the brain's network backbone. We found that the pivotal WM connections with highly topological-edge centrality were primarily distributed in several long-range cortico-cortical connections (including the corpus callosum, cingulum and inferior fronto-occipital fasciculus) and some projection tracts linking subcortical regions. These pivotal WM connections exhibited high levels of microstructural organization indicated by diffusion measures (the fractional anisotropy, the mean diffusivity and the axial diffusivity) and greater physical consumption indicated by streamline lengths, and contributed significantly to the brain's hubs and the rich-club structure. Network motif analysis further revealed their heavy participations in the organization of communication blocks, especially in routes involving inter-hemispheric heterotopic and extremely remote intra-hemispheric systems. Computational simulation models indicated the sharp decrease of global network integrity when attacking these highly centralized edges. Together, our results demonstrated high building-cost consumption and substantial communication capacity contributions for pivotal WM connections, which deepens our understanding of the topological mechanisms that govern the organization of human connectomes.

## Introduction

The white matter (WM) fibers, i.e., bundles of myelinated axons that provide connections between gray matter (GM) regions, serve as the foundation of information communication in the human brain. Their wiring patterns directly determine the topological performance of brain networks. With recent advances in diffusion magnetic resonance imaging (MRI) techniques and computational tractography methods (Mori et al., 1999), the WM fiber bundles in human brain can be reconstructed in a noninvasive way and the whole-brain structural networks can be generated at a macroscopic level (Hagmann et al., 2008; Gong et al., 2009), with nodes representing GM regions and edges representing the characteristics of WM fiber bundles linking GM regions. The mapping and descriptions of topological organization in human brain WM networks [i.e., “human connectomics” (Sporns et al., 2005)] has led to compelling discoveries of topological properties of brain networks, including their small-worldness (Hagmann et al., 2008; Gong et al., 2009), modular structure (Hagmann et al., 2008; He et al., 2009), highly connected hubs (Sporns et al., 2007; Hagmann et al., 2008; Gong et al., 2009; van den Heuvel and Sporns, 2013), and rich-club organization (van den Heuvel and Sporns, 2011; van den Heuvel et al., 2012). Compared to these global and nodal properties, the topological roles of network edges (i.e., WM tracts) that are responsible for information transfer across regions or systems have been less explored. Several long-range cortico-cortical WM connections, such as the corpus callosum, superior longitudinal fasciculus and cingulum, have been observed to be more frequently involved in efficient information transfer in the network than short-range ones (Gong et al., 2009). From an “edge-centric” perspective, de Reus et al. (2014) demonstrated the effects of the disrupted connections on network topologies and within different network communities. However, the underlying principles for the different topological roles of WM tracts are largely unknown.

What, in terms of wiring substrates and contributions, makes certain WM tracts more topologically important than others? Specifically, (i) do the macro-structural communication capacities of WM connections depend upon their microstructural organization and other physical consumptions such as fiber length? Heavier communicational/information loads flowing in these macro-scale brain networks may impose higher demands on biological resources, reflected by high-level microstructural properties (e.g., high axonal density or compact myelin) and long projection distance (Laughlin and Sejnowski, 2003; Kaiser and Hilgetag, 2006); (ii) How do the pivotal WM connections contribute to the topological properties of network hubs and rich-club structure? The WM tracts with high communication capacity could facilitate the transfer of the massive signals generated and processed by the biologically costly hubs (Vaishnavi et al., 2010; Liang et al., 2013; Tomasi et al., 2013; Collin et al., 2014), and therefore, contributing to the formation of the core structure such as rich-club. (iii) What are the path-motif patterns in the human brain structural networks in terms of pivotal edges? The minimum communication blocks, defined as the shortest paths between regions, indicate the simplest yet complete information routes in a brain network (Milo et al., 2002; van den Heuvel et al., 2012). Their patterns of utilization of pivotal edges, namely their path motifs, could reflect the information transfer strategy of the human brain.

To address these issues, we collected two scanning sessions of diffusion MRI data in 57 healthy adults and built up the human whole-brain structural networks. We first fully chart the pivotal WM connections, characterized by highly topological centralization, which then allow us to study their critical characteristics. The communication capacity of WM edges in the brain network was quantified using the edge-betweenness centrality measurement (Freeman, 1977; Girvan and Newman, 2002), and highly centralized WM connections were identified as pivotal edges, with their wiring substrates (including WM microstructural organization and streamline length) and topological nexus with brain hubs, rich-club and path motif profiles systematically examined. Validations were performed using two sessions of data, with both low- (90 nodes) and high-resolution (1024 nodes) nodal definitions.

## Materials and methods

### Participants

The data employed in this study were a subset of the Connectivity-based Brain Imaging Research Database (C-BIRD) at Beijing Normal University. This subset includes data from 57 participants (male/female: 30/27; age: 19–30 years) who completed two MRI scan sessions at an interval of approximately 6-weeks (40.94 ± 4.51 days). All participants were right-handed and had no history of neurological or psychiatric disorders. Written informed consent was obtained from each participant, and this study was approved by the Institutional Review Board of the State Key Laboratory of Cognitive Neuroscience and Learning at Beijing Normal University. These data have been released in the Consortium for Reliability and Reproducibility (CoRR) dataset (http://fcon_1000.projects.nitrc.org/indi/CoRR/html/bnu_1.html; Lin et al., 2015).

### Data acquisition and preprocessing

All MRI data were obtained using a Siemens Trio Tim 3.0 T scanner (Siemens Medical Systems, Erlangen, Germany) with a 12-channel phased-array head coil in the Imaging Center for Brain Research, Beijing Normal University. Diffusion weighted imaging data were acquired using a single-shot twice-refocused spin-echo diffusion echo-planar imaging sequence. The sequence parameters were repetition time (TR) = 8000 ms, echo time (TE) = 89 ms, 30 non-collinear diffusion directions with b = 1000 s/mm^2^ and an additional volume with b = 0 s/mm^2^, data matrix = 128 × 128, field of view (FOV) = 282 mm × 282 mm, 2.2 mm slice thickness, isotropic voxel size (2.2 mm^3^), bandwidth = 1562 Hz/pixel, and 62 transverse slices without gap covering the whole brain, number of excitation = 2. Three-dimensional high-resolution brain structural images were acquired by using a T1-weighted, sagittal 3D magnetization prepared rapid gradient echo (MP-RAGE) sequences. The sequence parameters were TR/TE/inversion time = 2530 ms/3.39 ms/1100 ms, flip angle = 7°, FOV = 256 mm × 256 mm, in-plane resolution = 256 × 256, slice thickness = 1.33 mm, and 144 sagittal slices covering the whole brain. The data of session 1 were used for the main analyses and the data of session 2 were used for validation analysis.

For each participant, the diffusion MRI data were preprocessed with FMRIB's Diffusion Toolbox of FSL (Version 5.0; http://www.fmrib.ox.ac.uk/fsl) to correct artifacts induced by head motion and eddy currents by applying an affine alignment of each diffusion-weighted image to the b0 image.

### Network construction

**Figure 1:**
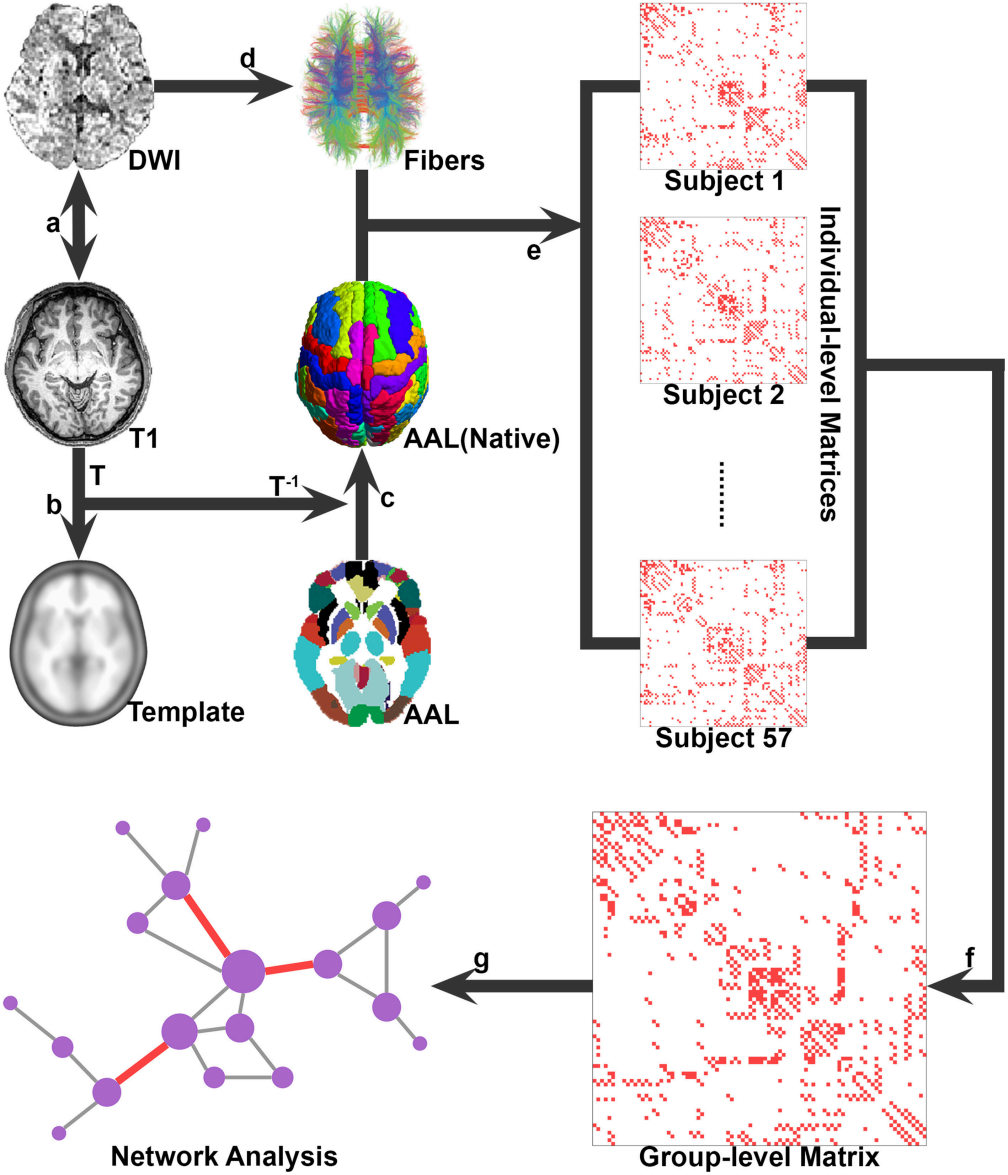
**A flowchart for the construction of the whole-brain WM network. (A)** The T1-weighted image was firstly rigidly coregistered to the averaged b0 image in native diffusion space; **(B)** The transformed T1 image was then nonlinearly transformed to the ICBM152 T1 template in the MNI space, and the transformation matrix T was estimated; **(C)** the inversed transformation T-1 was used to warp the AAL atlas from the MNI space to the native diffusion space, obtaining the parcellation for each individual; **(D)** the whole-brain WM tracts were reconstructed by using deterministic tractography; **(E)** the WM fibers connecting each pair of regions were determined for each subject, thus the individual WM networks were constructed; **(F)** the group-level connectivity matrix was computed by selecting all connections that were present in at least 50% of the group of individuals; and **(G)** both the individual and group-level networks were further analyzed by using graph theoretical methods.

Figure 1 illustrates the flowchart of the construction of whole-brain structural networks, which was outlined as follows.

#### Network node definition

In this study, we employed the Automated Anatomical Labeling Atlas (AAL) (Tzourio-Mazoyer et al., 2002) to parcel the brain into 90 cortical and subcortical regions. The detailed procedure for brain WM networks construction has been described previously (Gong et al., 2009). In brief, for each individual, the T1-weighted images were firstly coregistered to the averaged b0 image (two b0 images were obtained for each subject in this study) in native diffusion space using a linear transformation. The transformed T1-weighted images were then nonlinearly transformed to the ICBM152 T1 template in the Montreal Neurological Institute (MNI) space and the transformation matrices were estimated. Finally, the inversed transformation was used to warp the AAL atlases from the MNI space to the native diffusion space, therefore, obtaining the parcellation for each individual. Notably, discrete labeling values in the atlas were preserved by the use of a nearest-neighbor interpolation method. All of these linear and nonlinear mappings were implemented by using the SPM8 package (www.fil.ion.ucl.ac.uk/spm/software/spm8/).

#### White matter tractography

Reconstruction of the whole-brain WM tracts was performed using DTIstudio (version 3.0.3) based on the Fiber Assignment by Continuous Tracking (FACT) algorithm (Mori et al., 1999). Fiber tracking was computed by seeding each voxel with fractional anisotropy (FA) value greater than 0.2. Fiber tracking was stopped at voxels where FA < 0.2 or if the turning angle between adjacent steps was greater than 45°. All the fiber pathways in the brain were reconstructed using the deterministic tractography method.

#### Network edge definition

To perform the analyses on both the group and individual level, we defined network edges in two ways. For the group-level network, from the set of 57 individual connectivity matrices, a group-level connectivity matrix was computed by selecting all connections that were present in at least 50% of the group of individuals (de Reus and van den Heuvel, 2013a). For the individual networks, we selected a threshold value for the number of streamlines. Two regions were considered to be structurally connected if there are at least 3 streamlines with end points located in these two regions. All of these networks were unweighted. Given the high consistency between the results of group-level and individual level analyses, we mainly reported the results of group-level WM network and treated individual network analyses as a validation.

### Network analysis

Network analyses were carried out by using the Matlab BGL package (www.stanford.edu/~dgleich/programs/matlab_bgl/) and the GRETNA toolkits (Wang et al., 2015). The 3D visualizations for brain networks were generated using the BrainNet Viewer (Xia et al., 2013).

#### Identification of pivotal edges

We calculated the edge betweenness centrality (EBC) for each edge in the WM network. The betweenness of an edge, *B*${}_{i}^{edge}$, is a global centrality measures that captures the influence of an edge over information flow between other nodes in the network. *B*${}_{i}^{edge}$ is defined as the number of shortest paths between any pairs of other nodes that pass through the edge (Freeman, 1977; Girvan and Newman, 2002):

$$\begin{array}{l} B_{i}^{edge}=\underset{j\;\neq \;k}{\sum }\frac{\sigma_{j,k\left(i\right)}}{\sigma_{j,k}} \end{array}$$

where, *j* and *k* are any two nodes that are not linked directly with edge *i* in the network, σ_*j, k*_ is the total number of shortest paths between nodes *j* and *k* and σ_*j, k*(*i*)_ is the number of shortest paths between nodes *j* and *k* that pass through edge *i*. The normalized EBC was then calculated as follows:

$$\begin{array}{l} b_{i}^{edge}=\frac{B_{i}^{edge}-mean\left(B_{i}^{edge}\right)}{std\left(B_{i}^{edge}\right)} \end{array}$$

where, *mean*(*B*${}_{i}^{edge}$) and *std*(*B*${}_{i}^{edge}$) is the mean value and the standard deviation (SD) of EBC in each network, respectively. The edges with the largest normalized EBC (*b*${}_{i}^{edge}$ > 1, i.e., the EBC > 1 SD above mean) were identified as the pivotal edges in the networks. Additionally, to examine the EBC distribution of the brain networks, we used three possible forms: a power-law, *P*(*x*) ~ α*x*^β^; an exponential model *P*(*x*) ~ αexp(β*x*); and an exponentially truncated power-law, *P*(*x*) ~ α*x*^β^exp(*x*/γ). The cumulative distribution was used to reduce the effects of noise (Strogatz, 2001), and the goodness of fitting was tested using R^2^ values.

#### The wiring substrates of the pivotal edges

To assess whether the betweenness of an edge depends on its wiring basis, including the WM microstructural properties and streamline length, we calculated the correlations between EBC and each of the WM tract properties and further compared the differences in these measurements between pivotal and non-pivotal edges. Previous research has demonstrated that the microstructural properties of WM such as axonal membrane or myelin can influence the degree of diffusion anisotropy indicated by the indices of diffusion tensor imaging (Beaulieu, 2002). Here, four WM diffusion indices were measured to assess the WM microstructural properties, including the FA, the mean diffusivity (MD), the axial diffusivity (AD) and the radial diffusivity (RD) (Basser, 1995; Song et al., 2002). These four metrics estimate different aspects of diffusion properties of the WM tissues: i) FA expresses the degree of anisotropy of a diffusion process in a given voxel: $FA=\sqrt{\frac{3}{2}}\frac{\sqrt{{\left(\lambda_{1}-\tilde{\lambda }\right)}^{2}-{\left(\lambda_{2}-\tilde{\lambda }\right)}^{2}-{\left(\lambda_{3}-\tilde{\lambda }\right)}^{2}}}{\sqrt{\lambda_{1}^{2}\;+\lambda_{2}^{2}\;+\lambda_{3}^{2}}}$, where $\tilde{\lambda }=\left(\lambda_{1}\;+\;\lambda_{2}+\lambda_{3}\right)∕3$; ii) MD is often used to estimate the total level of diffusion: *MD* = (λ_1_ + λ_2_ + λ_3_)/3; iii) AD is a metric of the level of diffusion in the direction of the first eigenvector and of local fiber orientation: *AD* = λ_1_; and iv) RD is an estimation of the amount of diffusion in the perpendicular to the first eigenvector and of the level of myelination of WM: *RD* = (λ_2_ + λ_3_)/2. For each edge, these four metrics were estimated by averaging the values of the voxels that the streamlines passed through, respectively. The streamline length of each edge, which represents its wiring costs (Kaiser and Hilgetag, 2006; Bullmore and Sporns, 2012), was estimated from the average length of the interconnecting streamlines in each individual network. For the group-level network, all the above metrics were calculated by averaging the values over existing edges across all individuals. Subsequently, Spearman's correlations were used to analyze the correlations between EBC and each of these fiber properties across all edges in the networks. Furthermore, the differences in these fiber properties between pivotal and non-pivotal edges were determined by permutation tests. To evaluate the total performance and consumption of the pivotal edges, we further calculated the proportion of EBC and streamline length of the pivotal edges. This was done by summing up the EBC and streamline length of all the pivotal edges and then dividing them by the total EBC and streamline length of the whole brain, respectively. The proportion curves of EBC and streamline length were plotted to demonstrate whether the pivotal edges have over-average values, in which the x-axis represents the proportion of edges sequenced in EBC and the y-axis represents the accumulate proportion of EBC or streamline length. Furthermore, the cost-performance for each edge was estimated by dividing the EBC by the streamline length and was compared between pivotal and non-pivotal edges.

#### Contributions to the nodal centralities

Considering that the topological properties of nodes and edges are highly interdependent in the brain network, we examined the relationship between the betweenness of the edge and the average nodal centralities of the two nodes it links. These nodal properties included nodal degree, efficiency, and betweenness. The nodal degree *K*_*i*_ is a basic topological property, which is defined as the number of links connected to a node:

$$\begin{array}{l} K_{i}=\underset{j\in N}{\sum }a_{ij} \end{array}$$

where *a*_*ij*_ = 1 if a connection exists between node *i* and node *j* in the unweighted network. The nodal efficiency reflects the averaged communication capability of the given node to others, which is defined as the averaged reciprocal of the shortest path length from the given node to other nodes (Latora and Marchiori, 2001):

$$\begin{array}{l} E_{i}=\frac{\underset{j\in N,j\neq i}{\sum }L_{ij}^{-1}}{N-1} \end{array}$$

where *L*${}_{ij}^{-1}$ is the reciprocal of the shortest path length between nodes *i* and *j*. The definition for nodal betweenness *B*${}_{i}^{node}$ is similar to edge betweenness, which is defined as the number of shortest paths between pairs of other nodes that pass through the node (Freeman, 1977):

$$\begin{array}{l} B_{i}^{node}=\underset{j\neq k\neq i}{\sum }\rho_{j,k\left(i\right)} \end{array}$$

where ρ_*j, k*(*i*)_ is the number of shortest paths between nodes *j* and *k* that pass through node *i*. Spearman's correlations were adopted to analyze the correlations between EBC and nodal degree, efficiency and betweenness, respectively, across all edges in the networks. The differences on these nodal properties between pivotal and non-pivotal edges were further determined by using permutation tests.

#### Contributions of the pivotal edges to the rich-club structure

The rich-club structure in networks is present when the high-degree nodes of a network tend to be more densely connected among themselves than expected by *chance*, thus forming a core architecture in the brain network. Such a structure has recently been revealed not only in animal brains (Harriger et al., 2012; de Reus and van den Heuvel, 2013b) but also in the human brain (van den Heuvel and Sporns, 2011; Collin et al., 2014). To assess whether the edges belonging to the rich-club had higher EBC and whether the rich-club had more pivotal edges, we examined the rich-club architecture of the WM network. The rich-club coefficient Φ(*k*) was first calculated as follows:

$$\begin{array}{l} \Phi \left(k\right)=\frac{2E_{>k}}{N_{>k}\left(N_{>k}-1\right)} \end{array}$$

where *k* is the nodal degree to define hubs, *E*_>*k*_ is the number of links among these hubs, and *N*_>*k*_ is the number of hub nodes. Then, the Φ(*k*) was normalized to Φ_*norm*(*k*)_ by comparing to the mean Φ(*k*) of 1000 random networks of equal size and similar connectivity distribution:

$$\begin{array}{l} \Phi_{norm}\left(k\right)=\frac{\Phi \left(k\right)}{\Phi_{random}\left(k\right)} \end{array}$$

where Φ_*random*_(*k*) is the averaged rich-club coefficient over the 1000 random networks. An increasing normalized Φ_*norm*_(*k*) > 1 over a range of *k* reflects the existence of rich-club architecture in a network. In this study, we selected the *k* where the Φ_*norm*_(*k*) reached the peak value (*k* = 14) as the threshold for hub definition, which represented the most significant rich-club architecture (we also validated the results of other thresholds, e.g., *k* > 9, see validation results). Once the rich hub nodes were determined, the edges in the network can be divided into three categories according to the nodes they linked: (i) “rich-club connections” linking rich-club nodes, (ii) “feeder connections” linking rich-club nodes to non-rich-club nodes, and (iii) “local connections” linking non-rich-club nodes to each other (van den Heuvel et al., 2012). Finally, the differences in EBC and proportion of number/EBC of the pivotal edges among these three categories of connections were determined by using ANOVA with post-hoc permutation tests and Chi square tests, respectively.

#### Communication length and path motifs

To further assess the importance of pivotal edges in brain communication, we analyzed the communication length and path motifs of the WM network. Both metrics were based on the path length between any pair of nodes in the network. Firstly, all 4005 (*n* × (*n* − 1) ∕ 2, *n = 90*) unique shortest paths between all 90 nodes in the WM network were traced. Second, the communication length of each shortest path was calculated as the total streamline length of the edges that were used in the path. Subsequently, for each of the shortest paths, the streamline length spent on pivotal or non-pivotal edges was calculated. Finally, once aggregated across all shortest paths, several indices were calculated, including (i) the percentage of paths through pivotal edges, which was calculated by dividing the number of shortest paths passing through pivotal edges by the total number of shortest paths (i.e., 4005); (ii) the percentage of communication lengths of paths through pivotal edges, which was computed by dividing the sum of total streamline length of those shortest paths through pivotal edges by the sum of the total streamline length of all the shortest paths; and (iii) the percentage of communication length spent on the pivotal edges in paths through pivotal edges, which was defined as the ratio between the sum of streamline length pivotal edges of every shortest paths and the total streamline length of all the shortest paths walking through pivotal edges.

The ordered sequence of the pivotal or the non-pivotal edges on the routes of each shortest path were referred to as the “path motifs.” Therefore, six patterns of path motifs were identified, including the “N” (non-pivotal), “P” (pivotal), “N-P” (non-pivotal—pivotal), “N-P-N” (non-pivotal—pivotal—non-pivotal), “P-N-P” (pivotal—non-pivotal—pivotal), and “N-P-N-P” (non-pivotal—pivotal—non-pivotal—pivotal). Notably, these path motifs only represented the changes on edge types, but not the exact edge numbers on the path. For example, the “N-P-N” included paths with three edges (NPN), four edges (NNPN, NPPN, and NPNN) or more edges (e.g., NNNPN). Almost all paths could be classified into the six path motifs according to their sequences of edges along the path traveled. Subsequently, the distribution of path motifs was obtained by counting the numbers of the shortest paths in each motif pattern. To assess whether the frequency of each path motif in the WM network was at chance, we generated 1000 matched random networks, identified their pivotal edges, and calculated their path motif distributions. The Z score for each path motif was then computed by subtracting the mean value from the value of proportion in the real WM network and dividing by the standard deviation of those in random networks. Furthermore, we examined the proportions of various path motifs across brain systems to investigate if different brain systems communicated via different path motif patterns. Specifically, all brain regions were first classified into ten different brain systems (i.e., the frontal, parietal, temporal, occipital, and subcortical systems in each hemisphere). Thus, all paths could be allocated into 55 different groups, according to the positions of the two brain regions they connected. Then the proportion of the path motifs for each brain region pair could be measured. The 55 groups could be further classified into four categories, including within system, intra-hemispheric between systems, inter-hemispheric homotopic and heterotopic paths. Finally, a hierarchical clustering analysis was performed to determine whether the four categories had different patterns of path motifs. Briefly, a dissimilarity matrix was calculated by estimating the Euclidean distance between each pair of path groups, and agglomerative hierarchical cluster trees were generated based on the dissimilarity matrix with the weighted linkage agglomerative algorithm.

#### Vulnerability and network robustness

The vulnerability is widely used to quantitatively measure the damage on the network performance caused by the simulated failure of its elements (Costa et al., 2007). To calculate the vulnerability of an individual edge in the WM network, we removed the edges one by one from the network and calculated the changes in global efficiency of the resulting networks. To test the effects of pivotal edges and non-pivotal edges on network performance, we compared the vulnerability values of these two groups using a permutation test.

Network robustness, characterized by the degree of tolerance against random failures and targeted attacks, is usually associated with the stability of a complex network. Here, we investigated the robustness of the networks by the removals of edges (Kaiser et al., 2007; He et al., 2008). To address the random edge failure tolerance of the WM network, we first randomly removed one edge from the networks and then measured the changes in the global efficiency and the size of the largest connected component of the networks. Then, we continued to select and remove additional edges from the networks randomly and recomputed the two measures. To evaluate the targeted attack tolerance, we repeated the above processes but removed the edges with the greatest betweenness. Considering the dynamical compensation in the human brain, we recalculated the edge betweenness after each removal.

### Validation analysis

To evaluate the reproducibility of our findings, we validated our main findings via the following four procedures. Firstly, we parceled the whole brain using a randomly generated high-resolution template with 1024 nodes (Zalesky et al., 2010), and reconstructed the WM network. We repeated our analysis to determine whether our main findings are independent from node definitions. Secondly, we performed the analysis on each of the individual WM networks to assess the reproducibility of those main findings on the individual level. Thirdly, we analyzed the imaging data of the same individuals scanned after an interval of approximately 41 days to determine whether there were consistent topological organizations of the WM network across two scans. Finally, to assess whether our group-level findings are sensitive to network construction thresholds, two additional thresholds were applied. We reconstructed the group-level networks by selecting all connections that were present in at least 40 or 60% of the group of individuals.

All of the comparisons between pivotal and non-pivotal edges were performed using permutation tests. Briefly, for each metric, we initially calculated the difference of the mean values between pivotal and non-pivotal edges. An empirical distribution of the difference was then obtained by randomly reallocating all of the values into two groups and re-computing the mean differences between the two randomized groups (10,000 permutations). The original difference between pivotal and non-pivotal edges was assigned a *p* value as the proportion of random values in the obtained empirical distribution. The 95th percentile points of the empirical distribution were used as critical values in a one-tailed test of whether the observed group differences could occur by chance.

### Results

We used diffusion MRI tractography approaches to construct the brain networks at both the group and the individual levels (Figure 1). The group-based brain network with 90 nodes was fully connected, with 431 WM connections and a connection density of 10.8%. For the individuals, the densities of the brain networks ranged from 8 to 12% (mean ± SD: 9.7% ± 0.9%). We further identified the pivotal WM connections (i.e., highly centralized edges) from the brain networks and systematically examined their physical characteristics and topological contributions to network communications. Given compatible results between 90-node and 1024-node networks and between the group- and individual-level networks, we mainly report the findings from the group-based network analyses with 90 nodes, unless specifically mentioned.

### Edge betweenness and pivotal edges in the WM networks

#### Edge betweenness distribution

**Figure 2:**
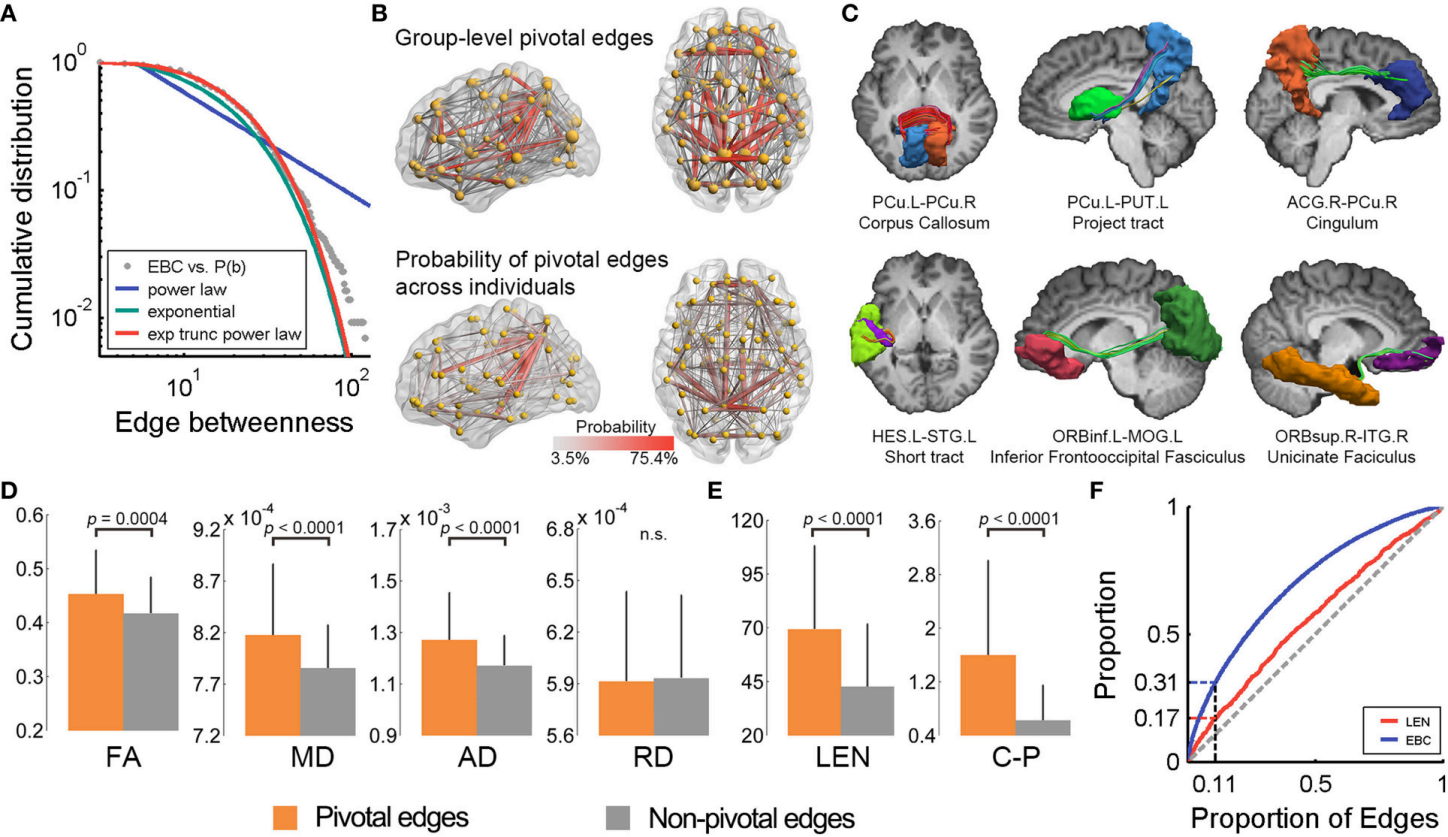
**The pivotal edges and their wiring substrates in the human WM network. (A)** The EBC distribution of the WM network was best fitted by an exponentially truncated power-law form. **(B)** The spatial pattern of the pivotal edges (red) of the group-level WM network (upper) is quite similar to the probability map of the pivotal edges across individuals. **(C)** Several pivotal WM edges were manifest in one representative subject. **(D)** The pivotal edges showed significantly higher levels of WM microstructural organization, as indicated by FA, MD, and AD, but not RD, than the non-pivotal ones. The error bars represent the standard deviation. **(E)** The pivotal edges also had greater streamline length and better cost-performance than the non-pivotal ones. **(F)** The curves for the proportion of edges vs. proportions of EBC and streamline length. EBC, edge betweenness centrality; exp, exponential; trunc, truncated; PCu, precuneus; PUT, putamen; ACG, anterior cingulate and paracingulate gyri; HES, Heschl's gyrus; STG, superior temporal gyrus; ORBinf, inferior frontal gyrus, orbital part; MOG, middle occipital gyrus; ORBsup, superior frontal gyrus, orbital part; ITG, inferior temporal gyrus; FA, fractional anisotropy; MD, mean diffusivity; AD, axial diffusivity; RD, radial diffusivity; LEN, streamline length; C-P, cost-performance; n.s., not significant; L, left; R, right.

We used the EBC (Freeman, 1977; Girvan and Newman, 2002) to quantify the topological centrality or communication capacity of the WM edges in the structural brain network. The EBC of an edge measures the frequency with which the shortest path between any region pair passes through this edge. The EBC distribution of the network was best fitted by the exponentially truncated power-law form [*P*(*x*) ~ αx^β^exp(*x*/γ)] rather than the power-law [*P*(*x*) ~ αx^β^] and or exponential [*P*(*x*) ~ αexp(βx)] models (Figure 2A). The estimated parameters were: α = 0.98, β = 0.20, γ = −15.42 and *R*^2^ = 0.998, respectively. Such a model indicates that i) the WM edges play heterogeneous roles in information communication across the network, and ii) the brain network includes some highly centralized edges but prevents the existence of extremely centralized WM connections with overly heavy loads.

#### Identifying pivotal edges

The WM edges with higher EBC (>1 SD above the whole-brain mean) are referred to as pivotal edges in the present study. Forty-eight of the 431 edges (11.1%) were identified as pivotal edges in the whole-brain WM network (Figure 2B and Table 1), including 16 inter-hemispheric and 32 intra-hemispheric (22 inter- and 10 intra-lobe) connections. The spatial distribution pattern of the group-based pivotal edges was largely consistent with the probability map of pivotal WM edges across individuals (Figure 2B). Specifically, these pivotal edges were primarily located in human major WM tracts, involving the corpus callosum (16/48), the cingulum (7/48), the uncinate fasciculus (4/48), and several projection tracts linking the subcortical with cortical regions (5/48) (Figure 2C and Table 1).

**Table 1:** **The pivotal edges of the human brain WM network and their properties**.

**No**.	**Region A**	**Region B**	**Normalized EBC**	**Fiber Length (mm)**	**Class**	**Lobes**	**WM tracts**	**Connection category**	**Vulnerability (%)**
1	PoCG.R	PCu.L	5.33	114.84	InterHemi	LP-RP	CC	Feeder	0.333
2	PCu.L	STG.L	5.26	87.92	InterLobe	LP-LT	Short tract	Feeder	0.242
3	PCu.L	PUT.L	4.93	88.92	InterLobe	LP-LS	Projection tract	Rich-club	0.212
4	ORBsup.L	ORBsup.R	4.79	86.84	InterHemi	LF-RF	CC	Rich-club	0.287
5	PCu.R	STG.R	3.75	87.45	InterLobe	RP-RT	Short tract	Feeder	0.248
6	PCu.R	PUT.R	3.73	81.81	InterLobe	RP-RS	Projection tract	Rich-club	0.250
7	HIP.L	HIP.R	3.53	92.38	InterHemi	LT-RT	CC	Local	0.395
8	SFGdor.R	IFGtriang.L	3.36	98.42	InterHemi	LF-RF	CC	Feeder	0.220
9	HES.L	STG.L	3.29	18.61	IntraLobe	LT-LT	Short tract	Local	1.505
10	HES.R	STG.R	3.29	14.82	IntraLobe	RT-RT	Short tract	Local	1.456
11	SPG.L	SPG.R	3.17	114.43	InterHemi	LP-RP	CC	Local	0.153
12	SPG.R	PCu.L	2.92	111.02	InterHemi	LP-RP	CC	Feeder	0.102
13	ORBsup.R	ITG.R	2.72	76.77	InterLobe	RF-RT	UF	Feeder	0.240
14	SOG.R	MOG.L	2.62	137.84	InterHemi	LR-RO	CC	Rich-club	0.162
15	CAL.R	PCu.L	2.53	68.02	InterHemi	LP-RO	CC	Rich-club	0.156
16	SPG.L	PCu.R	2.42	105.99	InterHemi	LP-RP	CC	Feeder	0.139
17	PCu.L	MTG.L	2.35	90.80	InterLobe	LP-LT	Cingulum	Feeder	0.111
18	PHG.L	PCu.L	2.25	39.17	InterLobe	LP-LT	Cingulum	Feeder	0.199
19	SFGdor.R	ORBsup.R	2.14	13.82	IntraLobe	RF-RF	Short tract	Rich-club	0.149
20	ACG.L	PCu.L	2.03	75.95	InterLobe	LF-LP	Cingulum	Feeder	0.218
21	MOG.L	PUT.L	1.84	89.45	IntraLobe	LO-LS	IFO	Rich-club	0.107
22	SFGdor.L	SFGdor.R	1.78	89.09	InterHemi	LF-RF	CC	Feeder	0.065
23	ORBsup.R	TPOmid.R	1.78	70.34	InterLobe	RF-RT	UF	Feeder	0.111
24	PCu.L	PCL.L	1.70	13.62	IntraLobe	LP-LP	Short tract	Feeder	0.172
25	HIP.R	THA.R	1.70	40.31	InterLobe	RT-RS	Undefined	Local	0.196
26	CAL.L	PCu.R	1.67	63.70	InterHemi	RP-LO	CC	Rich-club	0.143
27	PreCG.R	PCL.L	1.65	125.33	InterHemi	RF-LP	CC	Local	0.142
28	PCu.L	THA.L	1.63	70.58	InterLobe	LP-LS	Projection tract	Feeder	0.102
29	DCG.L	PCu.L	1.60	33.52	InterLobe	LF-LP	Cingulum	Feeder	0.176
30	ORBsup.R	PUT.R	1.59	38.59	InterLobe	RF-RS	Projection tract	Rich-club	0.097
31	PHG.R	PCu.R	1.57	40.99	InterLobe	RP-RT	Cingulum	Feeder	0.121
32	DCG.R	PCu.R	1.55	32.99	InterLobe	RF-RP	Cingulum	Feeder	0.190
33	ORBsup.L	ITG.L	1.54	70.46	InterLobe	LF-LT	UF	Feeder	0.149
34	PCu.L	PCu.R	1.53	96.65	InterHemi	LP-RP	CC	Rich-club	0.056
35	ACG.R	PCu.R	1.48	84.79	InterLobe	RF-RP	Cingulum	Feeder	0.181
36	CAU.L	PUT.L	1.42	11.21	IntraLobe	LS-LS	Undefined	Feeder	0.116
37	SOG.R	PCu.L	1.40	93.38	InterHemi	LP-RO	CC	Rich-club	0.079
38	ORBinf.L	MOG.L	1.38	137.25	InterLobe	LF-RO	IFO	Feeder	0.158
39	PUT.R	PAL.R	1.34	12.63	IntraLobe	RS-RS	Undefined	Feeder	0.133
40	PCu.L	PCL.R	1.30	111.80	InterHemi	LP-RP	CC	Feeder	0.218
41	SFGdor.R	PUT.R	1.28	48.90	InterLobe	RF-RS	Projection tract	Rich-club	0.074
42	PCG.R	PCu.L	1.20	33.89	InterHemi	LP-RP	CC	Feeder	0.162
43	ORBinf.R	CAL.R	1.17	144.23	InterLobe	RF-RO	IFO	Feeder	0.123
44	ORBsup.L	INS.L	1.12	18.42	InterLobe	LF-LS	UF	Feeder	0.134
45	PreCG.R	MTG.R	1.11	95.09	InterLobe	RF-RT	Short tract	Local	0.128
46	CAU.L	THA.L	1.07	25.22	IntraLobe	LS-LS	Undefined	Local	0.116
47	PUT.L	PAL.L	1.06	12.18	IntraLobe	LS-LS	Undefined	Feeder	0.102
48	CAU.R	PUT.R	1.04	11.82	IntraLobe	RS-RS	Undefined	Feeder	0.097

Fiber tracts were determined by examining the tractography result of each individual with prior anatomical knowledge. No., number; EBC, edge betweenness centrality; PoCG, postcentral gyrus; R, right; PCu, precuneus; L, left; ORBsup, superior frontal gyrus, orbital part; HIP, hippocampus; SFGdor, superior frontal gyrus, dorsolateral; HES, Heschl gyrus; SPG, superior parietal gyrus; SOG, superior occipital gyrus; CAL, calcarine fissure and surrounding cortex; PHG, parahippocampal gyrus; ACG, anterior cingulate and paracingulate gyri; PreCG, precentral gyrus; DCG, median cingulate and paracingulate gyri; CAU, caudate nucleus; ORBinf, inferior frontal gyrus, orbital part; PCG, posterior cingulate gyrus; PUT, putamen; STG, superior temporal gyrus; IFGtriang, inferior frontal gyrus, triangular part; ITG, inferior temporal gyrus; MOG, middle occipital gyrus; MTG, middle temporal gyrus; TPOmid, temporal pole: middle temporal gyrus; PCL, paracentral lobule; THA, thalamus; INS, insula; InterHemi, inter-hemispheric connection; InterLobe; intra-hemispheric inter-lobe connection; IntraLobe, within lobe connections; LP, left parietal lobe; RP, right parietal lobe; LT, left temporal lobe; LS, left subcortical; LF, left frontal lobe; RF, right frontal lobe; RT, right temporal lobe; RS, right subcortical; RO, right occipital; LO, left occipital; CC, corpus callosum; UF, uncinate fasciculus; IFO, inferior frontooccipital fasciculus.

#### Pivotal edges within and between brain systems

We examined the spatial layout of the pivotal edges across different brain systems (frontal, parietal, temporal, occipital and subcortical): (i) While classifying all WM edges into three categories (i.e., inter-hemispheric, intra-hemispheric between- and within-systems), both the EBC values and proportion of pivotal edges were significantly different [EBC: *F*_(2, 428)_ = 21.3, *p* = 1.9 × 10^−9^; Proportion: χ^2^_(2)_ = 22.3, *p* = 1.0 × 10^−5^], with a descending order of inter-hemispheric, between- and within-system connections; (ii) The parietal regions had the greatest proportion of pivotal connections (50%, 24/48) (Figures 2B,C), with 45.8% (22/48) of the pivotal edges structurally connected with the bilateral precuneus, the frequently reported structural cores in previous human connectome studies (Hagmann et al., 2008; Gong et al., 2009; van den Heuvel and Sporns, 2011).

### The wiring substrates of the pivotal WM edges

We further explored whether topologically centralized network edges involve high-level microstructural organization and expensive physical consumption. The WM diffusion properties, including the FA, MD, and AD, exhibited significantly higher values in the pivotal edges compared to the non-pivotal ones (all *p*s < 0.0004, permutation tests) (Figure 2D). Notably, the RD did not show significant difference in EBC between the pivotal and non-pivotal edges (*p* = 0.41, permutation test). The pivotal WM edges exhibited significantly greater streamline lengths than the non-pivotal ones (*p* < 0.0001, permutation test) (Figure 2E), and the EBC was positively correlated with streamline length across edges (Spearman ρ = 0.29, *p* < 0.0001) (Table S1), suggesting that these centrally embedded WM connections tend to span longer physical distances. The ratio of EBC to streamline length, quantifying the communication capacity per unit of streamline length or cost-performance, was significantly higher in the pivotal edges than the non-pivotal edges (*p* < 0.0001, permutation test) (Figure 2E). Lastly, we charted the curves for the proportion of edges vs. proportions of EBC and streamline length, and found that the pivotal edges (10.8% in number) consumed 16.9% of the streamline length but contributed 31.3% to the total communication capacity (in terms of EBC) of the whole brain (Figure 2F). These results together suggest the costly but highly cost-efficient signature of the pivotal edges.

### Contributions of the pivotal edges to the network hubs/rich-club structure

#### Contribution to the nodal properties

**Figure 3:**
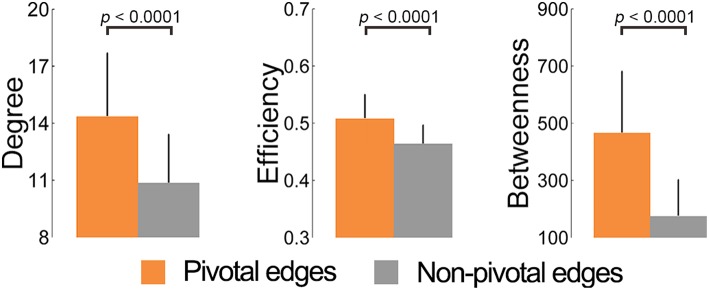
**Contribution of the pivotal edges toward the centrality of the nodes they linked**. The pivotal edges had significantly greater contributions to all three nodal properties (nodal degree, efficiency, and betweenness) than the non-pivotal ones. The contribution toward nodal centralities of an edge was estimated by averaging nodal properties of its two linking nodes.

We explored the relationship between the EBC values of WM edges and their linked nodes' properties (nodal degree, efficiency, and betweenness). The pivotal edges had significantly greater contributions to all three nodal properties than the non-pivotal ones (all *p*s < 0.0001, permutation tests) (Figure 3). Moreover, significant positive correlations were found over all three nodal centralities and across all edges, with the Spearman's correlation coefficients of 0.35, 0.38, and 0.59, respectively (all *p*s < 0.0001) (Table S1). These results indicated a strong topological nexus between pivotal edges and pivotal nodes in the WM network.

#### Contribution to the rich-club architecture

**Figure 4:**
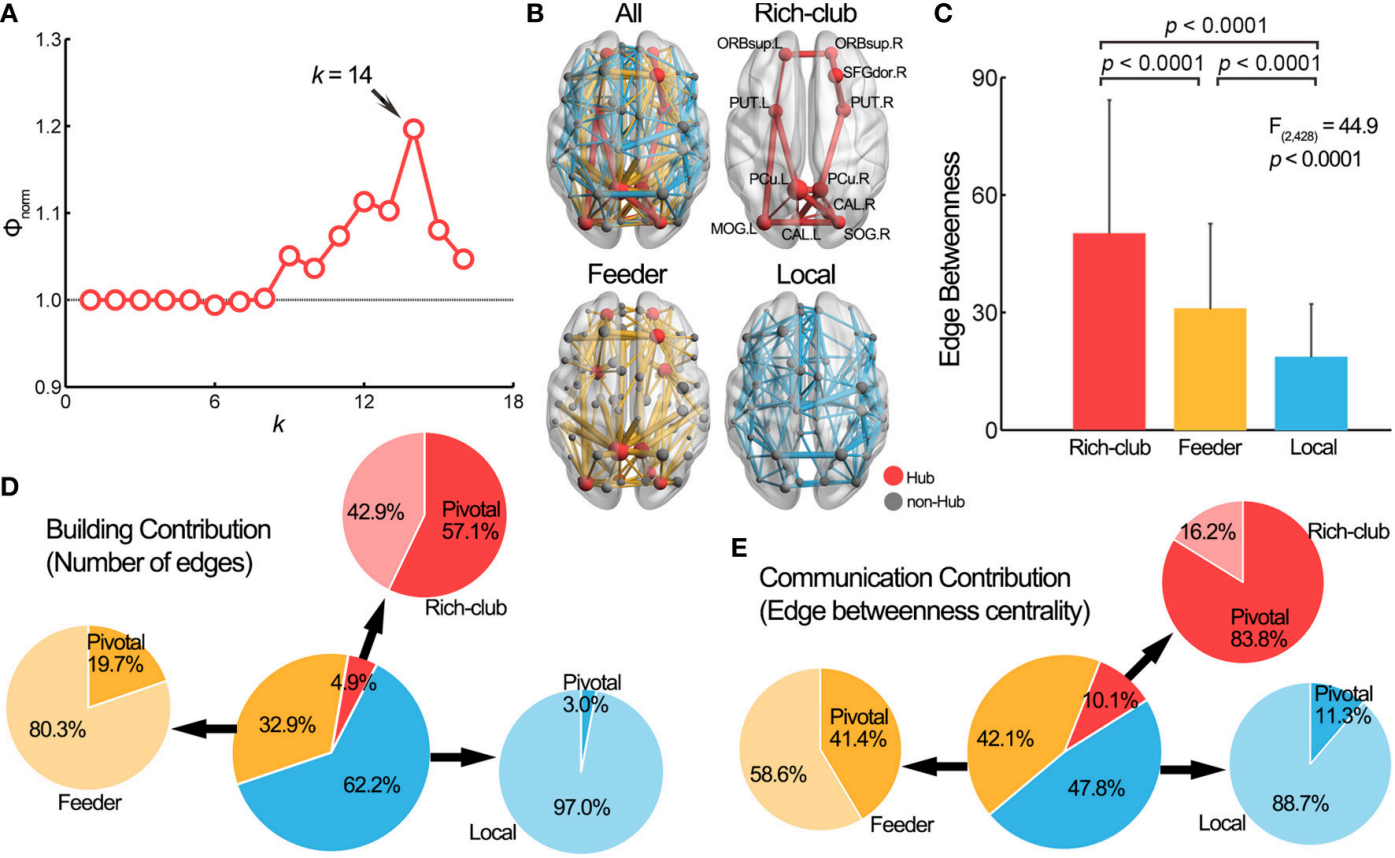
**Pivotal edges and the rich-club structure. (A)** The normalized rich-club coefficient Φ_*norm*_(*k*) of the group-level WM network was above 1 for a range of *k* from 9 to 16. The peak at *k* = 14 was selected as the hub threshold for further analysis. **(B)** The network hubs were mainly located in the medial line of the brain and the connections of the brain network can be further classified into three categories: rich-club (red), feeder (yellow) and local (blue) connections. **(C)** The edge betweenness centrality values were significantly different among rich-club, feeder, and local connections. **(D)** The pivotal edges had significantly different building contribution (indicated by the proportion of number) to three categories of connections. **(E)** The communication contribution (indicated by the proportion of edge betweenness centrality), of the pivotal edges was also significantly different among three categories of connections. The center pie illustrates the building/communication percentage of the three types of connections, and the surrounding pies show the building/communication percentage in each category of the connections. SFGdor, superior frontal gyrus, dorsolateral; CAL, Calcarine fissure and surrounding cortex; L, left; R, right. For other abbreviations, see Figure 2.

We examined the contribution of pivotal WM edges to the rich-club architecture of the WM network. Figure 4A illustrates the curve of the normalized rich-club coefficient, Φ_*norm*_(*k*), over a range of nodal degree, *k*. The Φ_*norm*_(*k*) were larger than 1 at *k* > 8, indicating the existence of a rich-club structure in the WM network (van den Heuvel and Sporns, 2011). Here, we chose the peak Φ_*norm*_(*k*), where the nodes with *k* > 14 were considered the brain hubs (see Table S2 for results of other thresholds). We identified 11 network hubs that were primarily located in the bilateral precuneus, the bilateral orbital part of superior frontal gyrus, the right dorsolateral superior frontal gyrus, the bilateral calcarine sulcus, the left middle occipital gyrus, the right superior occipital gyrus and the bilateral putamen (Figure 4B). Most of these hubs (72.7%) were structurally connected with the pivotal edges. Further, we divided the whole-brain WM connections into three categories according to the types of nodes they linked (van den Heuvel et al., 2012): rich-club connections between rich-club nodes (*n* = 21), feeder connections between rich-club and non-rich-club nodes (*n* = 142) and local connections between two non-rich-club nodes (*n* = 268). Significant differences in EBC were observed among these three edge categories [*F*_(2, 428)_ = 44.9, *p* = 2.0 × 10^−18^], with a descending order of the rich-club, feeder and local connections (post hoc comparisons, permutation tests, all *p*s < 0.001) (Figure 4C). Importantly, the proportions of the number of pivotal WM edges among the three categories, which represents the network building contribution, were significantly different [$\chi_{\left(2\right)}^{2}=73.5$, *p* = 1.1 × 10^−16^]: 57.1% (12/21) within the rich-club connections, 19.7% (28/142) within the feeder connections and 3.0% (8/268) within the local connections (Figure 4D). The proportion of EBC of the pivotal edges among the three categories, which represents the network communication contribution, was also significantly different [$\chi_{\left(2\right)}^{2}$ = 2490.3, *p* < 1.0 × 10^−64^]: 83.8% within the rich-club connections, 41.4% within the feeder connections and 11.3% within the local connections (Figure 4E). These results suggest that the rich-club architecture of the brain networks was topologically supported by the pivotal WM edges.

### Communication length and path motifs

#### Communication length of pivotal edges

When investigating the shortest paths (i.e., the minimum communication block) between any two nodes in the brain network, 58% of the paths (4005 in total) were found to pass through the pivotal edges. The pivotal-edge related paths accounted for 66% of the total communication length (communication length of one shortest path was defined as the total streamline length of the edges along the path). Specifically, when considering only the pivotal edge related paths, the total streamline length of these pivotal edges accounted for 67% of the total communication length, suggesting their high utilizations in brain communication.

#### Path motifs of the WM network

**Figure 5:**
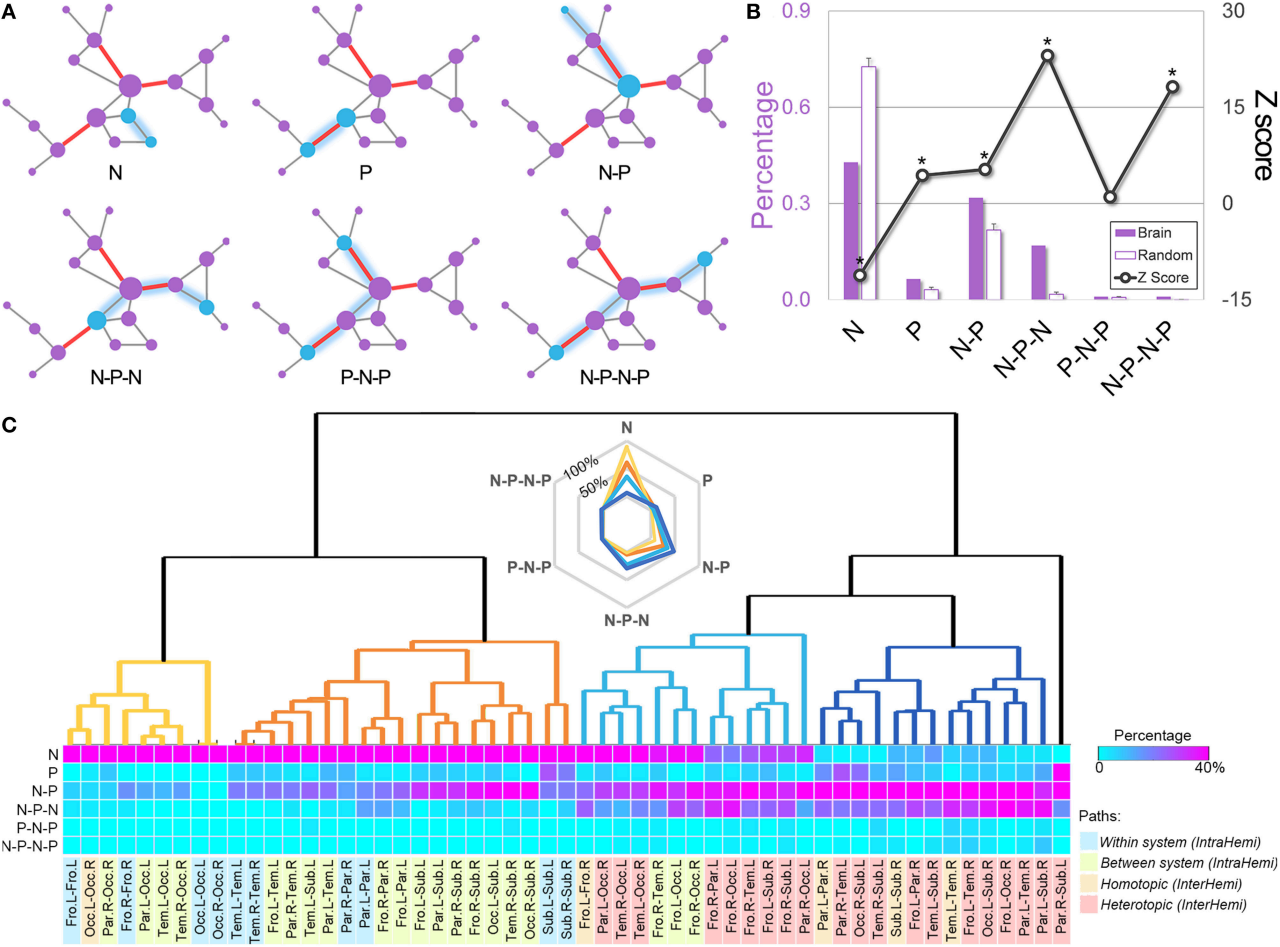
**Path motifs of the whole-brain WM network and of different brain systems. (A)** Examples of the six types of path motifs in the whole-brain WM network. Path motifs were defined as the ordered sequence of the pivotal or the non-pivotal edges on the routes of each shortest path. N, non-pivotal edge; P, pivotal edge. **(B)** The frequency percentage and normalized distribution of path motifs derived by comparing the actual frequency of each path motif to that of 1000 equivalent random networks. The “non-pivotal to pivotal to non-pivotal” (N-P-N) path motif was the most frequent path motif in the brain network (*Z* = 23.1). **(C)** The bottom matrix shows the proportions of path motifs between each pair of the brain systems. The following hierarchical clustering analysis revealed four path-motif distribution patterns, of which the path motif “N” decreased gradually while the “P” related path motifs constantly accumulated. Notably, most within-system and intra hemispheric paths communicated with the style “N,” while the inter-hemispheric paths, especially paths between heterotopic systems, utilized the pivotal edges more often. Fro, frontal; L, left; Occ, occipital; R, right; Par, parietal; Tem, temporal; Sub, subcortical; IntraHemi, intra hemispheric; InterHemi, inter hemispheric.

Every shortest path between nodes walks through a series of edges, of which the ordered sequence of the pivotal or the ordinary edges on the routes was referred to as the “path motifs.” Six types of path motif were identified in the whole-brain WM network (Figure 5A), and their appearing frequencies were statistically compared to those of 1000 equivalent random networks. The comparison revealed that the paths in the brain network exhibited significantly greater percentages of several pivotal-edge related connection types (e.g., “P,” “N-P,” “N-P-N,” and “N-P-N-P,” all Zs > 4.4, Figure 5B), especially the non-pivotal to pivotal to non-pivotal (N-P-N) path motif (*Z* = 23.1). Such a path-motif distribution profile indicates the central role of pivotal edges in the communicational organization of brain circuits.

#### Path motifs across different brain systems

Hierarchical clustering analysis of the path motifs across different brain systems revealed four distinct patterns, with gradual changes from the topologically simplest path motif “N” to complex “P” related patterns (Figure 5C): (i) the paths within the frontal and occipital systems in each hemisphere, and several occipital related intra-hemispheric paths mostly travel through the non-pivotal edges (mean percentage of “N”: 89.2%), indicating a plainest communication pattern between these regions; (ii) the pattern with a moderate percentage of motif “N” (61.0%) and a small percentage of motif “N-P” (26.3%) was observed in the paths within the parietal, temporal and subcortical systems, and in most of the intra-hemispheric paths; (iii) the pattern with fewer motif “N” (35.8%) but more “P” related motifs [57.3%, including “N-P” (34.8%) and “N-P-N” (22.5%)] were primarily distributed in the homotopic paths between bilateral frontal systems and heterotopic paths related to frontal and occipital systems; and iv) the last pattern with mostly the “P” related motifs [88.4%, including “P” (11.6%), “N-P” (47.5%), and “N-P-N” (29.3%)] primarily existed in the homotopic and heterotopic paths among bilateral parietal, temporal and subcortical regions. These findings suggest that the path motifs are distinctively differentiated in their communication across different brain systems: the heterotopic systems between hemispheres and extremely distant intra-hemispheric systems (e.g., between frontal and occipital systems) tend to adopt the pivotal related paths, while other intra-hemispheric systems tend to use plain communication patterns.

### Lesion simulations

**Figure 6:**
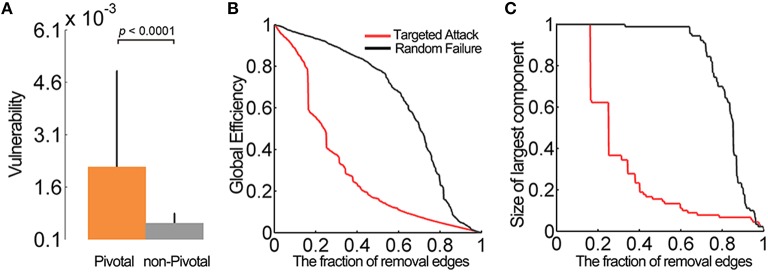
**Vulnerability of the pivotal edges and lesion simulation. (A)** The pivotal edges showed significant greater vulnerability than the non-pivotal ones. The error bars represent the standard deviation. **(B)** The global efficiency and **(C)** the size of largest connected component slowly declined in the random failure. When facing the targeted attacks, these network properties decreased rapidly (over 40%) after the top 20% edges were removed.

Two simulation strategies were used to evaluate how a “lesion” of the pivotal WM edges influenced brain network performance. First, we calculated the vulnerability of each edge in the WM network, which was defined as the change in the global efficiency of the network after eliminating this edge from the whole-brain network (Costa et al., 2007). Not surprisingly, the pivotal edges showed significantly greater vulnerability than the non-pivotal ones (*p* < 0.0001, permutation tests) (Figure 6A), especially the precuneus-related fiber tracts (Table 1). Second, we evaluated the topological robustness of the brain networks against random failure and targeted attacks of WM edges (Kaiser et al., 2007; He et al., 2008). Both the global efficiency and the size of the largest-connected component slowly declined in response to the random failure; In contrast, these network properties decreased rapidly in response to a targeted attack, with an over 40% reduction when 20% of the most centralized edges were attacked (Figures 6B,C). These simulation analyses highlight the topological significance of the pivotal WM edges in the global integrity of brain networks.

### Validation results

We evaluated the reproducibility of our main findings in several different ways, involving thresholds for rich-club, high-resolution brain parcellations, analyzing individual WM networks, data from another session and different connectivity thresholds during network constructions. We found the main results remained unchanged (Supplemental Results, Figures S1–S3, Tables S2–S6), indicating a robust reliability of our findings.

## Discussion

We mapped the small proportion of WM connections in human whole-brain networks that were highly topologically centralized in terms of global brain communication. These pivotal WM connections exhibited higher levels of WM microstructural organization and consumed longer streamline lengths, while they topologically contributed significantly to the brain's hub and rich-club architecture as well as the communication blocks, especially in the routes between inter-hemispheric and extremely remote intra-hemispheric systems. Simulation models showed that the integrity of brain networks would decrease sharply when pivotal edges were under attack. These signatures of pivotal WM connections were highly reproducible across individuals, scan sessions, spatial scales and network construction strategies.

### Spatial distribution of pivotal WM connections

We found that different WM tracts play heterogeneous roles in global information communication in brain networks. The most centralized WM connections were primarily located in several major long-range WM pathways, such as the corpus callosum, cingulum and inferior fronto-occipital fasciculus, which are the vital communication lines across regions to support human cognition. For instance, one third of the pivotal edges belonging to the inter-hemispheric connections located in the corpus callosum, which coordinates numerous functional integrations processes including perception, attention, memory, language and reasoning (Gazzaniga, 2000). The cingulum that connects medial frontal, parietal and temporal systems, is the principal route of the default mode network (Greicius et al., 2009), damage to which is associated with various cognitive impairments (Metzler-Baddeley et al., 2012). Several projection tracts are also topologically centralized, involving fibers linking the subcortical nuclei such as the putamen, the thalamus and the caudate nuclei, which are hubs of the human WM networks (van den Heuvel and Sporns, 2011; Crossley et al., 2014) and are involved in various brain functions, (Burgess et al., 2002; Packard and Knowlton, 2002; Kunimatsu and Tanaka, 2010). From an anatomic embedding perspective, these WM tracts, especially the posterior cerebral WM, have also been demonstrated to be more involved in the construction of the brain network as the voxels within these regions had more network edges passing through them (Owen et al., 2015). Together, these pivotal WM connections provide critical high-throughput communication channels or shortcuts between cortical and subcortical regions.

### The WM microstructural organization and wiring cost of the pivotal WM connections

The pivotal WM connections showed higher levels of microstructural organization, as indicated by greater FA, MD, and AD values than non-pivotal ones, which was mainly driven by the higher AD value in pivotal WM tracts. Such results might reflect more orderly fiber organization, greater axonal diameter, larger packing densities, higher proportion of myelinated axons of these pivotal edges (Basser, 1995; Beaulieu, 2002). The pivotal edges also showed greater physical consumption, indicated by streamline length, suggesting an extraordinary wiring cost for building these topologically centralized shortcuts that facilitate the link across distant brain regions. Intriguingly, the unit communication capacity (dividing EBC by streamline length) is higher for pivotal edges, indicating their rewarding building cost. Together, the high-level microstructural organization and longer axonal fibers empower these pivotal edges to have faster communication routes with shorter transmission delays, which consequently facilitate synchronous information processing, increase signal transfer robustness and reduce noise during communication (Laughlin and Sejnowski, 2003; Kaiser and Hilgetag, 2006; Collin et al., 2014). This phenomenon, wherein a small number of high-quality and long-range pivotal edges consume substantial resources to ensure high levels of both local and global information integration of the brain, provides additional support for the concept of cost-efficiency balance of neural circuitry formation (Bullmore and Sporns, 2012).

### Contributions of the pivotal edges toward the network hubs/rich-club architectures

The pivotal edges had a significantly greater contribution to nodal topological properties than the non-pivotal ones. Biologically costly hubs are supposed to generate and process massive signals to achieve the functional integration (Sporns et al., 2007; Buckner et al., 2009; Liang et al., 2013; Tomasi et al., 2013; van den Heuvel and Sporns, 2013). Nearly 80% of pivotal WM connections were directly linked with hubs. These pivotal WM connections with high-throughout transfer capability could fulfill the communicational requirement of the hubs and further nourish their developments. In fact, a significant positive correlation between nodal centrality and the anatomical distance of edges has been reported (Alexander-Bloch et al., 2013; Crossley et al., 2014). The associations between the edge-betweenness centrality and nodal centralities observed here provide more direct evidences for the interactive nexus between pivotal edges and brain hubs. Furthermore, approximately 20% of the pivotal WM edges connect non-hub regions. Such organization profiles avoid the over-aggregation of pivotal WM connections and formation of dominating clusters that shoulders extremely large communication loads. While clusters with extremely huge loads could be consumptive and vulnerable to pathogenic processes, the distributed localization of pivotal edges may help launch a compensation mechanism in cases of disease.

Recent studies have identified significantly denser connections between hub regions compared with non-hub regions, forming a rich-club core architecture in the structural brain networks in humans (van den Heuvel and Sporns, 2011) and other species (Harriger et al., 2012; de Reus and van den Heuvel, 2013b; Towlson et al., 2013). The rich-club organization is biologically costly in terms of metabolism and wiring cost but provides functional benefits by enhancing both the global information flow and the resilience of the network to hub attacks (van den Heuvel and Sporns, 2011; van den Heuvel et al., 2012). Here, from a perspective of network edges, we showed significant contributions of the pivotal WM connections to either the building number or the communication load within the rich-club architecture, suggesting that the pivotal WM connections facilitate the communication in the rich-club. Notably, some pivotal connections were those feeder connections linking hubs and non-hubs, reflecting their key roles in guiding and shunting of information flow around the communication cores.

### Utilization of the pivotal WM connections in communication strategy

One particularly intriguing finding here is that the communication blocks (i.e., the shortest paths) in the brain network are mainly associated with pivotal edges: the popular communication motif follows a route sequence of edges, where signals pass through increasingly centralized edges and then decreasing centrality (following an “N-P-N” path motif), indicating the pattern of first traveling on a side road, then turning onto highways, and finally leaving the arterial traffic. Interestingly, van den Heuvel et al. (2012) examined the relationship between the shortest communication paths and the rich-club architecture, and revealed a “zooming-out/zooming-in” structure of shortest paths by which the signals fed into, traversed, and exited the rich-club. They contended that this pattern is an expression of the degree-based “greedy routing” (Kleinberg, 2000) navigation strategy, an efficient communication scheme in technology and transportation networks in which the travel paths are selected only on the basis of local information without the knowledge of the global topography of the network (Simsek and Jensen, 2008; Boguna et al., 2009). Information about the global brain structure is probably absent from the local neurons or brain regions; therefore, sending information to distant targets by traveling through the topologically centralized WM connections for their spreading possibility could be an optimal path. Notably, we also observed diverse communication path motifs across different brain systems: inter-hemispheric paths communicating brain systems are more dependent on pivotal WM connections than intra-hemispheric within- or between-system paths; path motifs of the parietal or subcortical regions are far more complex than those of the systems with homogeneous and/or primary functions (e.g., occipital). These patterns inspire new questions about exactly how specific path motifs are related to the types of functions of a particular system.

In summary, our findings provide crucial evidence for the mechanisms underlying efficient communication strategies in human WM networks: investing in a “highway” system and utilizing it in the support of hubs, rich-club structures, and most-prevalent path-motifs. Such findings demonstrate a specific manifestation of the cost-efficiency principle in brain WM networks and deepen the understanding of signal exchange patterns across regions.

### Limitations and further considerations

Several methodological limitations warrant further consideration. First, diffusion tensor imaging in associated with inaccuracies in resolving crossing fibers and sharp angulations of tracts (Wedeen et al., 2008), which could lead to false-negatives in tracing for long-range fibers and false-positive connections between nearby regions. We applied a higher FA threshold (0.2) in fiber tracing to minimize possible artifacts due to the acquisition and tractography noise. However, the unbalanced numbers of long- and short-range connections might overestimate the importance of long-range connections, and result in the increase of FA of pivotal edges due to the fact that longer edges pass through high myelinated central white matter tracts. Future studies could be conducted by collecting diffusion spectrum imaging or high angular resolution diffusion imaging and reconstructing WM pathways with complex geometries, despite of the noise due to the high b-values (i.e., strong gradient) and long acquisition times. Second, the diffusion properties, including the FA, MD, AD, and RD, were estimated to represent the microstructural organization of the pivotal edges. Although these metrics provide approximate reflections of specific microstructural attributes such as fiber organization, axon density and myelination, the accurate biological interpretation for these indices remains ambiguous and controversial (Jones et al., 2013). Future studies using advanced diffusion MR models such as CHARMED (Assaf et al., 2004; Assaf and Basser, 2005), AxCaliber (Assaf et al., 2008), and ActiveAx (Alexander et al., 2010), and other MR contrast mechanisms such as quantitative magnetization transfer imaging (Sled and Pike, 2001; Cercignani and Alexander, 2006) might provide more accurate microstructural information. Third, relating the functional characteristics (e.g., dynamics and causality) (Hiltunen et al., 2014; Zalesky et al., 2014) of pivotal edges in functional networks to the pivotal WM connections is important for understanding the possible mechanisms of structural and functional coupling. Fourth, incorporating computational modeling approaches (Honey et al., 2009; Raj et al., 2012; Vertes et al., 2012; Deco et al., 2014) into the analysis of pivotal WM connections can further emulate their topological properties in an empirical situation. Conversely, optimizing the model parameters by taking into account the physical and topological characters of pivotal WM connections might help design artificial networks. Finally, a simulation “lesion” analysis was performed here to evaluate the topological influences of the pivotal edges on global brain communication. Real disease data are desirable to ascertain how brain lesions located in pivotal WM connections affect the topological performance of brain networks and subsequently cognitive dysfunctions.

## Author contributions

MX, YB, and YH: designed research; MX, QL, YB, and YH: performed research; MX, QL, and YH: contributed analytic tools; MX, QL, and YH: analyzed data; and MX, YB, and YH wrote the paper.

### Conflict of interest statement

The authors declare that the research was conducted in the absence of any commercial or financial relationships that could be construed as a potential conflict of interest. The Reviewer OT, declares that, despite being affiliated with the same institution as the Associate Editor SN, the review process was handled objectively. The Review Editor PM, declares that, despite being affiliated with the same institution as the Associate Editor SN, the review process was handled objectively. The Reviewer JO, declares that, despite being affiliated with the same institution as the Associate Editor SN, the review process was handled objectively.

## References

[B1] Alexander D. C. Hubbard P. L. Hall M. G. Moore E. A. Ptito M. Parker G. J.. (2010). Orientationally invariant indices of axon diameter and density from diffusion MRI. Neuroimage 52, 1374–1389. 10.1016/j.neuroimage.2010.05.043 20580932

[B2] Alexander-Bloch A. F. Vertes P. E. Stidd R. Lalonde F. Clasen L. Rapoport J.. (2013). The anatomical distance of functional connections predicts brain network topology in health and schizophrenia. Cereb. Cortex 23, 127–138. 10.1093/cercor/bhr388 22275481 PMC3513955

[B3] Assaf Y. Basser P. J. (2005). Composite hindered and restricted model of diffusion (CHARMED) MR imaging of the human brain. Neuroimage 27, 48–58. 10.1016/j.neuroimage.2005.03.042 15979342

[B4] Assaf Y. Blumenfeld-Katzir T. Yovel Y. Basser P. J. (2008). AxCaliber: a method for measuring axon diameter distribution from diffusion MRI. Magn. Reson. Med. 59, 1347–1354. 10.1002/mrm.21577 18506799 PMC4667732

[B5] Assaf Y. Freidlin R. Z. Rohde G. K. Basser P. J. (2004). New modeling and experimental framework to characterize hindered and restricted water diffusion in brain white matter. Magn. Reson. Med. 52, 965–978. 10.1002/mrm.20274 15508168

[B6] Basser P. J. (1995). Inferring microstructural features and the physiological state of tissues from diffusion-weighted images. NMR Biomed. 8, 333–344. 10.1002/nbm.1940080707 8739270

[B7] Beaulieu C. (2002). The basis of anisotropic water diffusion in the nervous system-a technical review. NMR Biomed. 15, 435–455. 10.1002/nbm.782 12489094

[B8] Boguna M. Krioukov D. Claffy K. C. (2009). Navigability of complex networks. Nature Physics 5, 74–80. 10.1038/nphys1130

[B9] Buckner R. L. Sepulcre J. Talukdar T. Krienen F. M. Liu H. Hedden T.. (2009). Cortical hubs revealed by intrinsic functional connectivity: mapping, assessment of stability, and relation to Alzheimer's disease. J. Neurosci. 29, 1860–1873. 10.1523/JNEUROSCI.5062-08.2009 19211893 PMC2750039

[B10] Bullmore E. Sporns O. (2012). The economy of brain network organization. Nat. Rev. Neurosci. 13, 336–349. 10.1038/nrn3214 22498897

[B11] Burgess N. Maguire E. A. O'Keefe J. (2002). The human hippocampus and spatial and episodic memory. Neuron 35, 625–641. 10.1016/S0896-6273(02)00830-9 12194864

[B12] Cercignani M. Alexander D. C. (2006). Optimal acquisition schemes for *in vivo* quantitative magnetization transfer MRI. Magn. Reson. Med. 56, 803–810. 10.1002/mrm.21003 16902982

[B13] Collin G. Sporns O. Mandl R. C. van den Heuvel M. P. (2014). Structural and functional aspects relating to cost and benefit of rich club organization in the human cerebral cortex. Cereb. Cortex 24, 2258–2267. 10.1093/cercor/bht064 23551922 PMC4128699

[B14] Costa L. D. Rodrigues F. A. Travieso G. Boas P. R. V. (2007). Characterization of complex networks: a survey of measurements. Proc. Natl. Acad. Sci. U.S.A. 56, 167–242. 10.1080/00018730601170527

[B15] Crossley N. A. Mechelli A. Scott J. Carletti F. Fox P. T. McGuire P.. (2014). The hubs of the human connectome are generally implicated in the anatomy of brain disorders. Brain 137, 2382–2395. 10.1093/brain/awu132 25057133 PMC4107735

[B16] Deco G. McIntosh A. R. Shen K. Hutchison R. M. Menon R. S. Everling S.. (2014). Identification of optimal structural connectivity using functional connectivity and neural modeling. J. Neurosci. 34, 7910–7916. 10.1523/JNEUROSCI.4423-13.2014 24899713 PMC6608269

[B17] de Reus M. A. Saenger V. M. Kahn R. S. van den Heuvel M. P. (2014). An edge-centric perspective on the human connectome: link communities in the brain. Philos. Trans. R. Soc. Lond. B. Biol. Sci. 369:20130527. 10.1098/rstb.2013.0527 25180305 PMC4150302

[B18] de Reus M. A. van den Heuvel M. P. (2013a). Estimating false positives and negatives in brain networks. Neuroimage 70, 402–409. 10.1016/j.neuroimage.2012.12.066 23296185

[B19] de Reus M. A. van den Heuvel M. P. (2013b). Rich club organization and intermodule communication in the cat connectome. J. Neurosci. 33, 12929–12939. 10.1523/JNEUROSCI.1448-13.2013 23926249 PMC6619725

[B20] Freeman L. C. (1977). A set of measures of centrality based on betweenness. Sociometry 40, 35–41. 10.2307/3033543

[B21] Gazzaniga M. S. (2000). Cerebral specialization and interhemispheric communication: does the corpus callosum enable the human condition? Brain 123(Pt 7), 1293–1326. 10.1093/brain/123.7.1293 10869045

[B22] Girvan M. Newman M. E. (2002). Community structure in social and biological networks. Proc. Natl. Acad. Sci. U.S.A. 99, 7821–7826. 10.1073/pnas.122653799 12060727 PMC122977

[B23] Gong G. He Y. Concha L. Lebel C. Gross D. W. Evans A. C.. (2009). Mapping anatomical connectivity patterns of human cerebral cortex using *in vivo* diffusion tensor imaging tractography. Cereb. Cortex 19, 524–536. 10.1093/cercor/bhn102 18567609 PMC2722790

[B24] Greicius M. D. Supekar K. Menon V. Dougherty R. F. (2009). Resting-state functional connectivity reflects structural connectivity in the default mode network. Cereb. Cortex 19, 72–78. 10.1093/cercor/bhn059 18403396 PMC2605172

[B25] Hagmann P. Cammoun L. Gigandet X. Meuli R. Honey C. J. Wedeen V. J.. (2008). Mapping the structural core of human cerebral cortex. PLoS Biol. 6:e159. 10.1371/journal.pbio.0060159 18597554 PMC2443193

[B26] Harriger L. van den Heuvel M. P. Sporns O. (2012). Rich club organization of macaque cerebral cortex and its role in network communication. PLoS ONE 7:e46497. 10.1371/journal.pone.0046497 23029538 PMC3460908

[B27] He Y. Chen Z. Evans A. (2008). Structural insights into aberrant topological patterns of large-scale cortical networks in Alzheimer's disease. J. Neurosci. 28, 4756–4766. 10.1523/JNEUROSCI.0141-08.2008 18448652 PMC6670444

[B28] He Y. Wang J. Wang L. Chen Z. J. Yan C. Yang H.. (2009). Uncovering intrinsic modular organization of spontaneous brain activity in humans. PLoS ONE 4:e5226. 10.1371/journal.pone.0005226 19381298 PMC2668183

[B29] Hiltunen T. Kantola J. Abou Elseoud A. Lepola P. Suominen K. Starck T.. (2014). Infra-slow EEG fluctuations are correlated with resting-state network dynamics in fMRI. J. Neurosci. 34, 356–362. 10.1523/JNEUROSCI.0276-13.2014 24403137 PMC6608153

[B30] Honey C. J. Sporns O. Cammoun L. Gigandet X. Thiran J. P. Meuli R.. (2009). Predicting human resting-state functional connectivity from structural connectivity. Proc. Natl. Acad. Sci. U.S.A. 106, 2035–2040. 10.1073/pnas.0811168106 19188601 PMC2634800

[B31] Jones D. K. Knösche T. R. Turner R. (2013). White matter integrity, fiber count, and other fallacies: the do's and don'ts of diffusion MRI. Neuroimage 73, 239–254. 10.1016/j.neuroimage.2012.06.081 22846632

[B32] Kaiser M. Hilgetag C. C. (2006). Nonoptimal component placement, but short processing paths, due to long-distance projections in neural systems. PLoS Comput. Biol. 2:e95. 10.1371/journal.pcbi.0020095 16848638 PMC1513269

[B33] Kaiser M. Martin R. Andras P. Young M. P. (2007). Simulation of robustness against lesions of cortical networks. Eur. J. Neurosci. 25, 3185–3192. 10.1111/j.1460-9568.2007.05574.x 17561832

[B34] Kleinberg J. M. (2000). Navigation in a small world. Nature 406, 845. 10.1038/35022643 10972276

[B35] Kunimatsu J. Tanaka M. (2010). Roles of the primate motor thalamus in the generation of antisaccades. J. Neurosci. 30, 5108–5117. 10.1523/JNEUROSCI.0406-10.2010 20371831 PMC6632795

[B36] Latora V. Marchiori M. (2001). Efficient behavior of small-world networks. Phys. Rev. Lett. 87, 198701. 10.1103/PhysRevLett.87.198701 11690461

[B37] Laughlin S. B. Sejnowski T. J. (2003). Communication in neuronal networks. Science 301, 1870–1874. 10.1126/science.1089662 14512617 PMC2930149

[B38] Liang X. Zou Q. He Y. Yang Y. (2013). Coupling of functional connectivity and regional cerebral blood flow reveals a physiological basis for network hubs of the human brain. Proc. Natl. Acad. Sci. U.S.A. 110, 1929–1934. 10.1073/pnas.1214900110 23319644 PMC3562840

[B39] Lin Q. Dai Z. Xia M. Han Z. Huang R. Gong G.. (2015). A connectivity-based test-retest dataset of multi-modal magnetic resonance imaging in young healthy adults. Sci. Data 2, 150056. 10.1038/sdata.2015.56 26528395 PMC4623457

[B40] Metzler-Baddeley C. Jones D. K. Steventon J. Westacott L. Aggleton J. P. O'sullivan M. J. (2012). Cingulum microstructure predicts cognitive control in older age and mild cognitive impairment. J. Neurosci. 32, 17612–17619. 10.1523/JNEUROSCI.3299-12.2012 23223284 PMC6621654

[B41] Milo R. Shen-Orr S. Itzkovitz S. Kashtan N. Chklovskii D. Alon U. (2002). Network motifs: simple building blocks of complex networks. Science 298, 824–827. 10.1126/science.298.5594.824 12399590

[B42] Mori S. Crain B. J. Chacko V. P. van Zijl P. C. (1999). Three-dimensional tracking of axonal projections in the brain by magnetic resonance imaging. Ann. Neurol. 45, 265–269. 9989633 10.1002/1531-8249(199902)45:2<265::aid-ana21>3.0.co;2-3

[B43] Owen J. P. Chang Y. S. Mukherjee P. (2015). Edge density imaging: mapping the anatomic embedding of the structural connectome within the white matter of the human brain. Neuroimage 109, 402–417. 10.1016/j.neuroimage.2015.01.007 25592996 PMC4340739

[B44] Packard M. G. Knowlton B. J. (2002). Learning and memory functions of the Basal Ganglia. Ann. Rev. Neurosci. 25, 563–593. 10.1146/annurev.neuro.25.112701.142937 12052921

[B45] Raj A. Kuceyeski A. Weiner M. (2012). A network diffusion model of disease progression in dementia. Neuron 73, 1204–1215. 10.1016/j.neuron.2011.12.040 22445347 PMC3623298

[B46] Simsek O. Jensen D. (2008). Navigating networks by using homophily and degree. Proc. Natl. Acad. Sci. U.S.A. 105, 12758–12762. 10.1073/pnas.0800497105 18725637 PMC2522263

[B47] Sled J. G. Pike G. B. (2001). Quantitative imaging of magnetization transfer exchange and relaxation properties *in vivo* using MRI. Magn. Reson. Med. 46, 923–931. 10.1002/mrm.1278 11675644

[B48] Song S. K. Sun S. W. Ramsbottom M. J. Chang C. Russell J. Cross A. H. (2002). Dysmyelination revealed through MRI as increased radial (but unchanged axial) diffusion of water. Neuroimage 17, 1429–1436. 10.1006/nimg.2002.1267 12414282

[B49] Sporns O. Honey C. J. Kötter R. (2007). Identification and classification of hubs in brain networks. PLoS ONE 2:e1049. 10.1371/journal.pone.0001049 17940613 PMC2013941

[B50] Sporns O. Tononi G. Kötter R. (2005). The human connectome: a structural description of the human brain. PLoS Comput. Biol. 1:e42. 10.1371/journal.pcbi.0010042 16201007 PMC1239902

[B51] Strogatz S. H. (2001). Exploring complex networks. Nature 410, 268–276. 10.1038/35065725 11258382

[B52] Tomasi D. Wang G. J. Volkow N. D. (2013). Energetic cost of brain functional connectivity. Proc. Natl. Acad. Sci. U.S.A. 110, 13642–13647. 10.1073/pnas.1303346110 23898179 PMC3746878

[B53] Towlson E. K. Vértes P. E. Ahnert S. E. Schafer W. R. Bullmore E. T. (2013). The rich club of the C. Proc. Natl. Acad. Sci. U.S.A. 33, 6380–6387. 10.1523/JNEUROSCI.3784-12.2013 23575836 PMC4104292

[B54] Tzourio-Mazoyer N. Landeau B. Papathanassiou D. Crivello F. Etard O. Delcroix N.. (2002). Automated anatomical labeling of activations in SPM using a macroscopic anatomical parcellation of the MNI MRI single-subject brain. Neuroimage 15, 273–289. 10.1006/nimg.2001.0978 11771995

[B55] Vaishnavi S. N. Vlassenko A. G. Rundle M. M. Snyder A. Z. Mintun M. A. Raichle M. E. (2010). Regional aerobic glycolysis in the human brain. Proc. Natl. Acad. Sci. U.S.A. 107, 17757–17762. 10.1073/pnas.1010459107 20837536 PMC2955101

[B56] van den Heuvel M. P. Kahn R. S. Goñi J. Sporns O. (2012). High-cost, high-capacity backbone for global brain communication. Proc. Natl. Acad. Sci. U.S.A. 109, 11372–11377. 10.1073/pnas.1203593109 22711833 PMC3396547

[B57] van den Heuvel M. P. Sporns O. (2011). Rich-club organization of the human connectome. J. Neurosci. 31, 15775–15786. 10.1523/JNEUROSCI.3539-11.2011 22049421 PMC6623027

[B58] van den Heuvel M. P. Sporns O. (2013). Network hubs in the human brain. Trends Cogn. Sci. 17, 683–696. 10.1016/j.tics.2013.09.012 24231140

[B59] Vértes P. E. Alexander-Bloch A. F. Gogtay N. Giedd J. N. Rapoport J. L. Bullmore E. T. (2012). Simple models of human brain functional networks. Proc. Natl. Acad. Sci. U.S.A. 109, 5868–5873. 10.1073/pnas.1111738109 22467830 PMC3326510

[B60] Wang J. Wang X. Xia M. Liao X. Evans A. He Y. (2015). GRETNA: a graph theoretical network analysis toolbox for imaging connectomics. Front. Hum. Neurosci. 9:386. 10.3389/fnhum.2015.00458 26175682 PMC4485071

[B61] Wedeen V. J. Wang R. P. Schmahmann J. D. Benner T. Tseng W. Y. Dai G.. (2008). Diffusion spectrum magnetic resonance imaging (DSI) tractography of crossing fibers. Neuroimage 41, 1267–1277. 10.1016/j.neuroimage.2008.03.036 18495497

[B62] Xia M. Wang J. He Y. (2013). BrainNet Viewer: a network visualization tool for human brain connectomics. PLoS ONE 8:e68910. 10.1371/journal.pone.0068910 23861951 PMC3701683

[B63] Zalesky A. Fornito A. Cocchi L. Gollo L. L. Breakspear M. (2014). Time-resolved resting-state brain networks. Proc. Natl. Acad. Sci. U.S.A. 111, 10341–10346. 10.1073/pnas.1400181111 24982140 PMC4104861

[B64] Zalesky A. Fornito A. Harding I. H. Cocchi L. Yucel M. Pantelis C.. (2010). Whole-brain anatomical networks: does the choice of nodes matter? Neuroimage 50, 970–983. 10.1016/j.neuroimage.2009.12.027 20035887

