# Supplementary material for: Connectomic Insights into Topologically Centralized Network Edges and Relevant Motifs in the Human Brain
Source: Front Hum Neurosci. 2016 Apr 19;10:158. doi: 10.3389/fnhum.2016.00158 (PMC4835491; doi:10.3389/fnhum.2016.00158)
Supplement: Supplementary file 1 [file Presentation1.PDF]

Supplemental Information

Results

Validation results

Thresholds for rich-club. Although the number of hub nodes gradually decreased when the degree threshold for rich-club was increased (k = 9, 10, …, 16), the main findings remained unchanged across different thresholds. For instance, significant differences in EBC were observed among three edge categories (all $Fs_{(2,428)} > 6.9$, ps < 0.0012), with a descending order of the rich-club, feeder and local connections. The building proportion and communicational contribution of pivotal edges were also significantly different among these three connection categories (building contributions: all $\chi^2 s_{(2)} > 10.3$, ps < 0.0058; communication contribution: all $\chi^2 s_{(2)} > 520.8$, ps < 1×10^{-64}): the pivotal edges contributed significantly more to the rich-club architecture than to the feeder and local connections (Table S2).

High-resolution WM network. We examined the pivotal edges and relevant properties in the high-resolution WM network (1,024 nodes). The group-level connectivity matrix was constructed by selecting all connections that were present in at least 25% of the group of individuals, resulting in a WM network with 3,987 edges and a density of 0.76%. The EBC distribution was also fitted by the exponentially truncated power-law form ($R^2 = 0.997$), and 242 edges (6.1%) were identified as pivotal edges. Consistent with the results of the low-resolution (90 nodes) network analysis, the microstructural organization and WM fiber cost (i.e., FA, MD, AD, and streamline length), cost-performance, nodal contributions (i.e., nodal degree, betweenness and efficiency) and vulnerability of the pivotal edges were significantly greater than non-pivotal ones (all ps < 0.02, Table S3). The proportion of the number of pivotal WM edges among the three categories, which represents the network building contribution, was significantly different in the high-resolution network (27.6% vs. 7.0% vs. 1.7%, $\chi^2_{(2)} = 352.1$, $p < 1 \times 10^{-64}$), and the proportion of EBC of the pivotal edges among the three categories, which represents the network communication contribution, was also significantly different (73.6% vs. 40.0% vs. 16.2%, $\chi^2_{(2)} = 4.7 \times 10^5$, $p < 1 \times 10^{-64}$). Notably, the hubs were defined as the top 12.2% of nodes with the highest degree to keep the similar proportion to the low-resolution WM network. Furthermore, the number of path motifs 'N-P-N' and 'N-P-N-P' remained significantly larger in the high-resolution network than the equivalent random networks (both Zs > 11.5), which was consistent with the findings of the 90-node network. We also noticed that with the increase of the network size, several high order path motif, namely the 'N-P-N-P-N' and 'N-P-N-P-N-P', were additionally observed, to be extremely abundant (Z = 805.2 and Z = 67.5, respectively) comparing to equivalent random networks, suggesting the high order communication character of the high-resolution networks (Fig. S1 and Table S3).

Individual networks. We found a high consistency between the results of individual- and group-level networks. Briefly, the individual EBC patterns were highly similar to the group-level

network (mean EBC across all individuals vs. EBC of group-level network: Spearman $\rho = 0.84$, $p < 1 \times 10^{-64}$), with 49.4 ± 6.1 pivotal edges. The EBC distributions of the individual networks were also best fitted by the exponentially truncated power-law form (R^2: 0.9968 - 0.9995). For each individual network, the pivotal edges exhibited enhanced physical properties (i.e., FA, MD, and AD) and nodal contributions (i.e., nodal degree, betweenness, and efficiency) (Table S4) and were significantly more vulnerable to target attacks compared to the non-pivotal edges (all *p*s < 0.02). Likewise, the building proportion and communicational contribution of pivotal edges were significantly different among the rich-club, feeder and local connections (building proportion: rich-club vs. feeder vs. local: 36.0% vs. 19.3% vs. 5.3%, $\chi^2_{(2)} = 31.3 \pm 14.9$, all *p*s < 0.007 for 56 individuals and $p = 0.064$ for Subject 22; communicational contribution: rich-club vs. feeder vs. local: 52.1% vs. 33.2% vs. 13.37%, $\chi^2_{(2)} = 759.4 \pm 454.5$, all *p*s < 1×10^{-64} for 56 individuals and $p = 0.13$ for Subject 33). Notably, the hubs were defined as the top 12.2% of nodes with the highest degree for each individual, that the proportion was similar to the group-level WM network. Furthermore, the profile of path motifs each individual network was highly similar to that of group-level network (Spearman *ρ*s > 0.89, *p*s < 0.033 for 52 individuals, and Spearman *ρ*s > 0.77, *p*s < 0.10 for remaining 5 individuals) (Fig. S2).

Data scanned at session 2. We performed the identical analysis on the data of session 2. The constructed group-level WM network contained 430 edges, and the EBC patterns were highly similar between the two scans (Spearman's $\rho = 0.90$, $p < 1 \times 10^{-64}$). Other results involving the EBC distribution, the relationship between EBC and fiber physical and integration properties, rich-club and path motif analysis were also highly similar to those obtained in the first scan (Fig. S3 and Table S5). Additionally, we also evaluated the test-retest reliability of EBC between the two sessions by calculating the intra-class correlation coefficient (ICC) for each edge:

$$ICC = \frac{BMS - WMS}{BMS + (s-1)WMS}$$

where BMS (WMS) represent the between-individual (within-individual) mean square and s indicates the number of repeated measurements of EBC (here, $s = 2$). Notably, we limited the ICC calculation to those edges occurred in the group-level networks both of session 1 and 2. The mean ICC on EBC of these edges reached 0.334 and we found that over 37% (150 out of 405) of the edges showed a fair to excellent (>0.4) test-retest reliability

Threshold for constructing group-level network. We constructed the group-level connectivity matrix by selecting all connections that were present in at least 50% of the group of individuals. However, the threshold might potentially affect our main findings. Thus, two additional group-level networks were constructed by applying two different thresholds and were analyzed, respectively (T40 network by 40% and T60 network by 60%). Both the T40 and T60 networks were fully connected, with 526 (density: 13.1%) and 358 (density: 8.9%) WM connections, respectively. The EBC distributions of these two networks were best fitted by the exponentially

truncated power-law form (both R^2s > 0.997), and 69 (13.1%) and 34 (9.5%) edges were identified as pivotal edges, respectively. Consistent with the results of the main group-level network analysis, the microstructural organization and WM fiber cost (i.e., FA, MD, AD, and streamline length), cost-performance, nodal contributions (i.e., nodal degree, betweenness, and efficiency) and vulnerability of the pivotal edges were significantly greater than non-pivotal ones (all *p*s < 0.0005, Table S6) in these two networks. The proportion of the number of pivotal WM edges among the three categories, which represents the network building contribution, were significantly different (T40: 45.8% vs. 23.6% vs. 3.6%, $\chi^2_{(2)} = 65.7$, $p = 5.3 \times 10^{-64}$; T60: 50.0% vs. 16.3% vs. 3.2%, $\chi^2_{(2)} = 43.6$, $p = 3.5 \times 10^{-10}$), and the proportion of EBC of the pivotal edges among the three categories, which represents the network communication contribution, was also significantly different (T40: 73.1% vs. 43.2% vs. 14.3%, $\chi^2_{(2)} = 1461.9$, $p < 1 \times 10^{-64}$; T60: 77.7% vs. 40.2% vs. 11.4%, $\chi^2_{(2)} = 2117.7$, $p < 1 \times 10^{-64}$). Notably, the hubs were defined as the top 12(T40) or 10 (T60) nodes with the highest degree, and that the proportion was similar to the main group-level WM network. Furthermore, the number of path motifs ‘N-P-N’ and ‘N-P-N-P’ remained significantly larger in these two networks than in the equivalent random networks (all Zs > 7.8), which was consistent with the findings of the main group-level network. Additionally, we noticed that the pivotal edge had significantly lower radial diffusivity (p = 0.0085) than the non-pivotal ones in the T40 network, which was not found in the main group-level network (Table S6).

Tables

Table S1. Statistics for group-level WM network

	Spearman's correlation between EBC and edge/nodal properties, ρ (p values)	Differences in edge/nodal properties between pivotal and non-pivotal edges (p values)
Fractional anisotropy (FA)	0.21 (1.7×10^{-5})	0.04 (0.0004)
Mean diffusivity (MD)	0.12 (0.0081)	3.2×10^{-5} (<0.0001)
Axial diffusivity (AD)	0.19 (8.6×10^{-5})	1.0×10^{-4} (<0.0001)
Radial diffusivity (RD)	-0.089 (0.067)	-1.9×10^{-6} (0.41)
Streamline length	0.29 (1.5×10^{-9})	26.66 (<0.0001)
Nodal degree	0.35 (7.3×10^{-14})	3.5 (<0.0001)
Nodal efficiency	0.38 (4.0×10^{-64})	0.04 (<0.0001)
Nodal betweenness	0.59 ($<1.0 \times 10^{-64}$)	291.3 (<0.0001)

Note: ρ, Spearman's correlation coefficient; EBC, edge betweenness centrality.

Table S2. Pivotal edges in rich-club architectures under different thresholds

Threshold	Edge betweenness centrality					Building contribution					Communication contribution					Number of hubs
	R	F	L	$F_{(2,428)}$	*p*-value	R (P, N)	F (P, N)	L (P, N)	$\chi^2_{(2)}$	*p*-value	R (P, N)	F (P, N)	L (P, N)	$\chi^2_{(2)}$	*p*-value	
k > 9	27.2 ± 23.0	23.9 ± 16.6	16.7 ± 15.5	6.9	0.0012	42.4% (16.4%, 83.6%)	42.9% (8.7%, 91.4%)	14.6% (3.2%, 96.8%)	10.3	0.0058	47.6% (42.0%, 58.0%)	42.4% (22.7%, 77.4%)	10.1% (17.0%, 83.0%)	520.8	<0.0001	43
k > 10	30.2 ± 25.7	24.1 ± 15.8	16.8 ± 15.7	13.2	<0.0001	29.0% (21.6%, 78.4%)	49.0% (8.5%, 91.5%)	22.0% (3.2%, 96.8%)	21.4	<0.0001	36.1% (50.6%, 49.4%)	48.6% (21.4%, 78.6%)	15.3% (17.0%, 83.0%)	1,036.5	<0.0001	34
k > 11	37.0 ± 28.4	25.7 ± 17.7	16.9 ± 13.5	29.8	<0.0001	16.0% (31.9%, 68.1%)	46.9% (11.4%, 88.7%)	37.1% (1.9%, 98.1%)	43.9	<0.0001	24.4% (60.9%, 39.1%)	49.7% (27.8%, 72.2%)	25.8% (10.1%, 89.9%)	1,638.1	<0.0001	23
k > 12	40.0 ± 30.2	26.8 ± 18.5	17.4 ± 13.3	34.3	<0.0001	12.1% (34.6%, 65.4%)	43.6% (13.8%, 86.2%)	44.3% (2.1%, 97.9%)	46.1	<0.0001	19.9% (63.7%, 36.3%)	48.5% (32.3%, 67.7%)	31.8% (9.5%, 90.5%)	1,749.2	<0.0001	19
k > 13	44.4 ± 30.5	28.7 ± 20.6	17.8 ± 13.0	40.3	<0.0001	7.9% (44.1%, 55.9%)	40.0% (16.3%, 83.7%)	52.2% (2.2%, 97.8%)	60.0	<0.0001	14.5% (71.6%, 28.4%)	47.2% (36.8%, 63.2%)	38.4% (9.4%, 90.7%)	2,107.9	<0.0001	15
k > 14	50.2 ± 34.0	31.0 ± 21.7	18.6 ± 13.5	44.9	<0.0001	4.9% (57.1%, 43.9%)	33.0% (19.7%, 80.3%)	62.2% (3.0%, 97.0%)	73.5	<0.0001	10.1% (83.8%, 16.2%)	42.1% (41.4%, 58.6%)	47.8% (11.3%, 88.7%)	2,490.0	<0.0001	11
k > 15	69.0 ± 33.8	36.3 ± 24.2	19.9 ± 14.0	51.6	<0.0001	1.4% (83.3%, 16.7%)	22.5% (29.9%, 70.1%)	76.1% (4.3%, 95.7%)	81.7	<0.0001	4.0% (92.6%, 7.4%)	33.7% (54.5%, 45.6%)	62.3% (14.9%, 85.1%)	2,419.1	<0.0001	6
k > 16	58.0 ± 28.3	39.2 ± 26.6	20.1 ± 14.9	45.7	<0.0001	0.9% (75.0%, 25.0%)	19.7% (34.1%, 65.9%)	79.4% (4.7%, 95.3%)	76.3	<0.0001	2.2% (86.8%, 13.2%)	31.9% (60.1%, 39.9%)	65.9% (15.5%, 84.5%)	2,419.0	<0.0001	5

Note: the data of edge betweenness centrality in each edge category are presented as mean ± standard deviation; the data of building contribution are presented as the percentage of the number of edges in each category accounted for the total edge number and the numbers in the bracket represent the ratio of pivotal and non-pivotal edges in each category; the data of communication contribution are presented as the percentage of the total edge betweenness in each edge category accounted for the total edge betweenness of the whole-brain network and the numbers in the bracket represent the ratio of pivotal and non-pivotal edges in each category. R, rich-club connection; F, feeder connection; L, local connection; P, pivotal edge; N, non-pivotal edge.

Table S3. Statistics for high-resolution whole-brain WM network

	Spearman's correlation between EBC and edge/nodal properties *ρ* (*p* values)	Differences in edge/nodal properties between pivotal and non-pivotal edges (*p* values)
Fractional anisotropy (FA)	0.20 (5.1×10^{-36})	0.03 (<0.0001)
Mean diffusivity (MD)	0.10 (1.6×10^{-9})	2.7×10^{-5} (<0.0001)
Axial diffusivity (AD)	0.22 (4.5×10^{-43})	8.2×10^{-5} (<0.0001)
Radial diffusivity (RD)	-0.06 (4.7×10^{-4})	-5.5×10^{-7} (0.44)
Streamline length	0.15 (8.4×10^{-23})	10.9 (<0.0001)
Nodal degree	0.34 ($< 1.0 \times 10^{-64}$)	5.8 (<0.0001)
Nodal efficiency	0.37 ($< 1.0 \times 10^{-64}$)	0.04 (<0.0001)
Nodal betweenness	0.70 ($< 1.0 \times 10^{-64}$)	27676 (<0.0001)

Note: ρ, Spearman's correlation coefficient; EBC, edge betweenness centrality; WM, white matter.

Table S4. Statistics for individual-level WM networks

	Spearman's correlation between EBC and edge/nodal properties *ρ* (N)	Differences in edge/nodal properties between pivotal and non-pivotal edges (N)
Fractional anisotropy (FA)	0.17 ± 0.06 (52)	0.03 ± 0.01 (47)
Mean diffusivity (MD)	0.12 ± 0.06 (34)	$1.7 \times 10^{-5} \pm 1.2 \times 10^{-5}$ (27)
Axial diffusivity (AD)	0.19 ± 0.05 (55)	$6.6 \times 10^{-5} \pm 2.5 \times 10^{-5}$ (51)
Radial diffusivity (RD)	-0.07 ± 0.07 (19)	$-8.0 \times 10^{-6} \pm 1.2 \times 10^{-5}$ (14)
Streamline length	0.25 ± 0.04 (57)	23.65 ± 6.41 (57)
Nodal degree	0.34 ± 0.08 (57)	2.30 ± 0.79 (57)
Nodal efficiency	0.37 ± 0.08 (57)	0.03 ± 0.01 (57)
Nodal betweenness	0.60 ± 0.06 (57)	217.23 ± 64.83 (57)

Note: data is presented as mean ± standard deviation, N represents the number of individuals showing significant correlations or between-group differences. EBC, edge betweenness centrality. The significant level was set as $p < 0.05$.

Table S5. Statistics for data of session 2

	Spearman's correlation between EBC and edge/nodal properties *ρ* (*p* values)	Differences in edge/nodal properties between pivotal and non-pivotal edges (*p* values)
Fractional anisotropy (FA)	0.18 (1.7×10^{-4})	0.04 (<0.0001)
Mean diffusivity (MD)	0.16 (7.1×10^{-4})	2.6×10^{-5} (<0.0001)
Axial diffusivity (AD)	0.20 (4.0×10^{-5})	9.7×10^{-5} (<0.0001)
Radial diffusivity (RD)	-0.08 (0.1)	-9.2×10^{-6} (0.1)
Streamline length	0.28 (3.7×10^{-9})	29.68 (<0.0001)
Nodal degree	0.43 (2.0×10^{-20})	3.5 (<0.0001)
Nodal efficiency	0.45 (4.4×10^{-23})	0.04 (<0.0001)
Nodal betweenness	0.64 (9.3×10^{-52})	279.6 (<0.0001)

Note: ρ, Spearman's correlation coefficient; EBC, edge betweenness centrality.

Table S6. Statistics for whole-brain WM networks that were constructed by different thresholds

	Spearman's correlation between EBC and edge/nodal properties *ρ* (*p* value)	Differences in edge/nodal properties between pivotal and non-pivotal edges (*p* values)
**T40 network**		
Fractional anisotropy (FA)	0.24 (1.4×10^{-8})	0.05 (<0.0001)
Mean diffusivity (MD)	0.14 (0.0018)	2.8×10^{-5} (<0.0001)
Axial diffusivity (AD)	0.22 (6.3×10^{-7})	1.1×10^{-4} (<0.0001)
Radial diffusivity (RD)	-0.15 (7.8×10^{-4})	-1.5×10^{-4} (0.0085)
Streamline length	0.32 (2.3×10^{-14})	28.8 (<0.0001)
Nodal degree	0.36 (6.6×10^{-18})	3.4 (<0.0001)
Nodal efficiency	0.38 (1.9×10^{-19})	0.04 (<0.0001)
Nodal betweenness	0.55 (2.4×10^{-43})	196.6 (<0.0001)
**T60 network**		
Fractional anisotropy (FA)	0.16 (0.0033)	0.03 (0.005)
Mean diffusivity (MD)	0.17 (0.0015)	2.6×10^{-5} (0.0019)
Axial diffusivity (AD)	0.18 (7.1×10^{-4})	8.5×10^{-5} (0.0003)
Radial diffusivity (RD)	-0.04 (0.49)	-3.8×10^{-6} (0.31)
Streamline length	0.25 (2.1×10^{-6})	31.2 (<0.0001)
Nodal degree	0.41 (1.3×10^{-15})	3.7 (<0.0001)
Nodal efficiency	0.42 (1.6×10^{-16})	0.05 (<0.0001)
Nodal efficiency	0.42 (1.6×10^{-16})	0.05 (<0.0001)

Note: ρ, Spearman's correlation coefficient; EBC, edge betweenness centrality; WM, white matter; T40, network generated with a threshold of 40%; T60, network generated with a threshold of 60%.

Figures

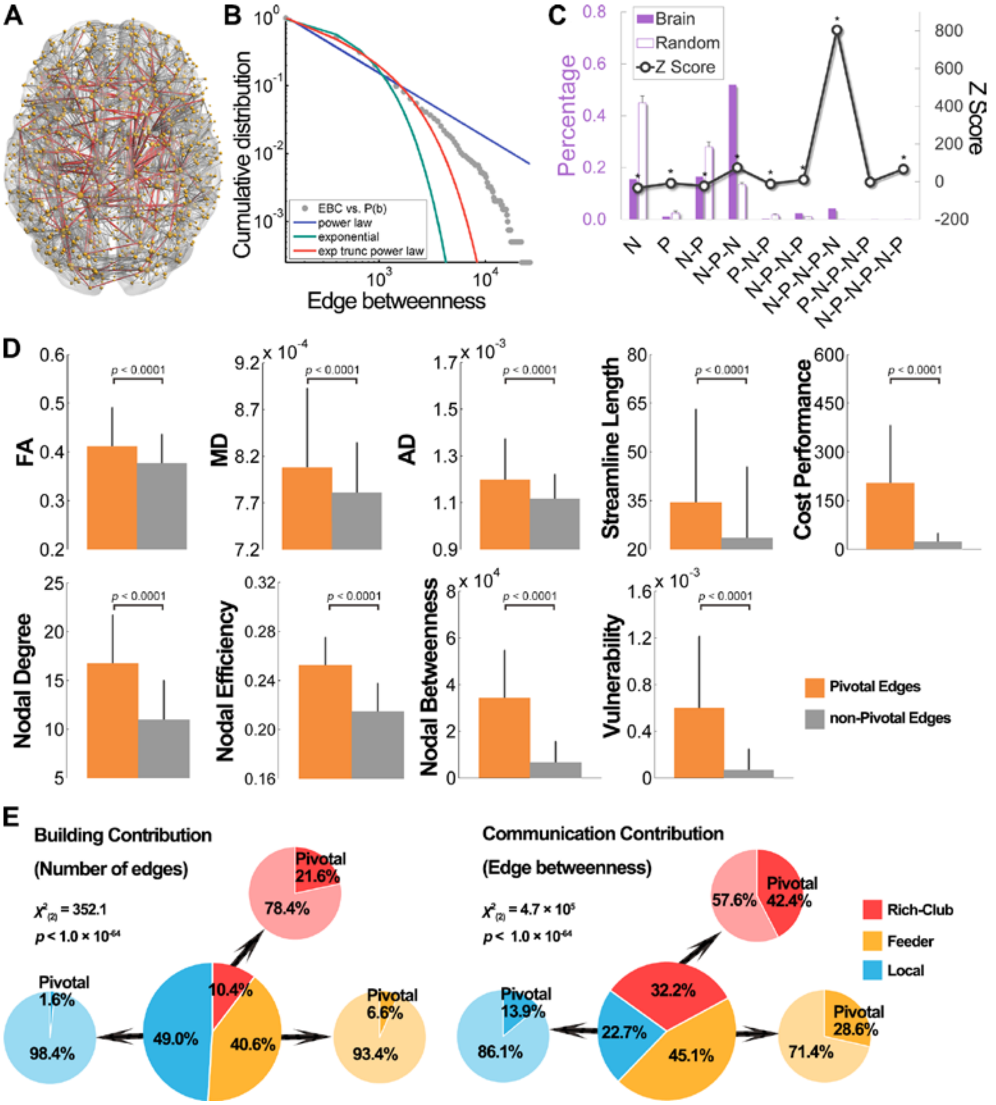

Figure S1. Validation on high resolution WM network. A) The 3D brain network manifested the pivotal edges (colored in red) of the high-resolution WM network were primarily connected with medial regions of the brain. **B)** The edge betweenness centrality distribution of the individual WM network was best fitted by an exponentially truncated power-law form (red, R^2 = 0.996) rather than power-law (blue, R^2 = 0.967) and exponential (green, R^2 = 0.985) models. **C)** The N-P-N-P-N and the N-P-N path motifs were the most two frequent path motif in the brain network (Z = 805.2 and Z = 74.6). **D)** The pivotal edges showed a significantly higher level of microstructural organization (as indicated by FA, MD and AD), longer streamline length, better cost-performance, greater vulnerability and more significant contributions to all three nodal properties (nodal degree, efficiency and betweenness, respectively) than the non-pivotal ones. The error bars represent the standard deviation. **E)** The building contribution (indicated by the proportion of number) and communication contribution (indicated by the proportion of edge betweenness centrality) of the pivotal edges were significantly different ($\chi^2_{(2)} = 352.1$, $p < 1.0 \times 10^{-64}$; $\chi^2_{(2)} = 4.7 \times 10^5$, $p < 1.0 \times 10^{-64}$) across three categories of connections. N, non-pivotal; P, pivotal; FA, fractional anisotropy; MD, mean diffusivity; AD, axial diffusivity.

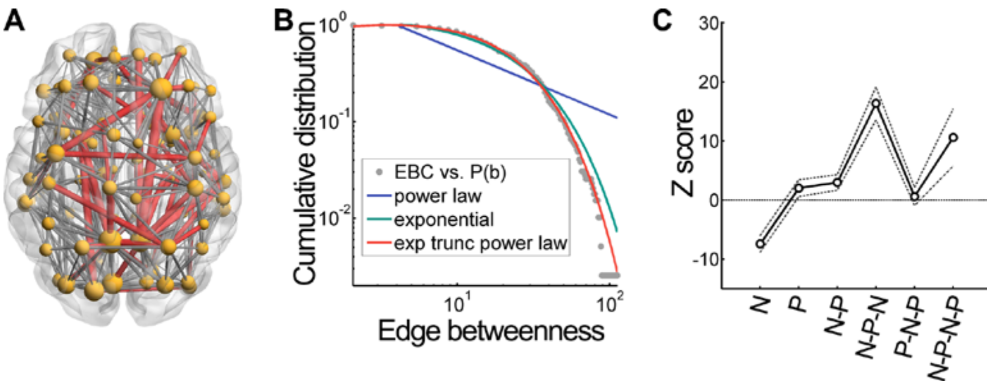

Figure S2. Validation in individual-level whole-brain WM networks. A) The spatial pattern of the pivotal edges (colored in red) of one representative subject was highly similar with the group-level network. The nodal size indicates the nodal degree, and the radius of the edges represents their edge betweenness centrality values. **B)** The edge betweenness centrality distribution of the individual WM network was best fitted by an exponentially truncated power-law form [$P(x) \sim \alpha x^{\beta} \exp(x/\gamma)$] (red, $R^2 = 0.999$) rather than power-law [$P(x) \sim \alpha x^{\beta}$] (blue, $R^2 = 0.748$) and exponential [$P(x) \sim \alpha \exp(\beta x)$] (green, $R^2 = 0.989$) models. **C)** The path motif profile of all individual whole-brain WM networks were largely similar with that of the group-level network. The solid line represents the mean Z-score and the dot lines indicate the standard deviation of the Z-score across all individuals.

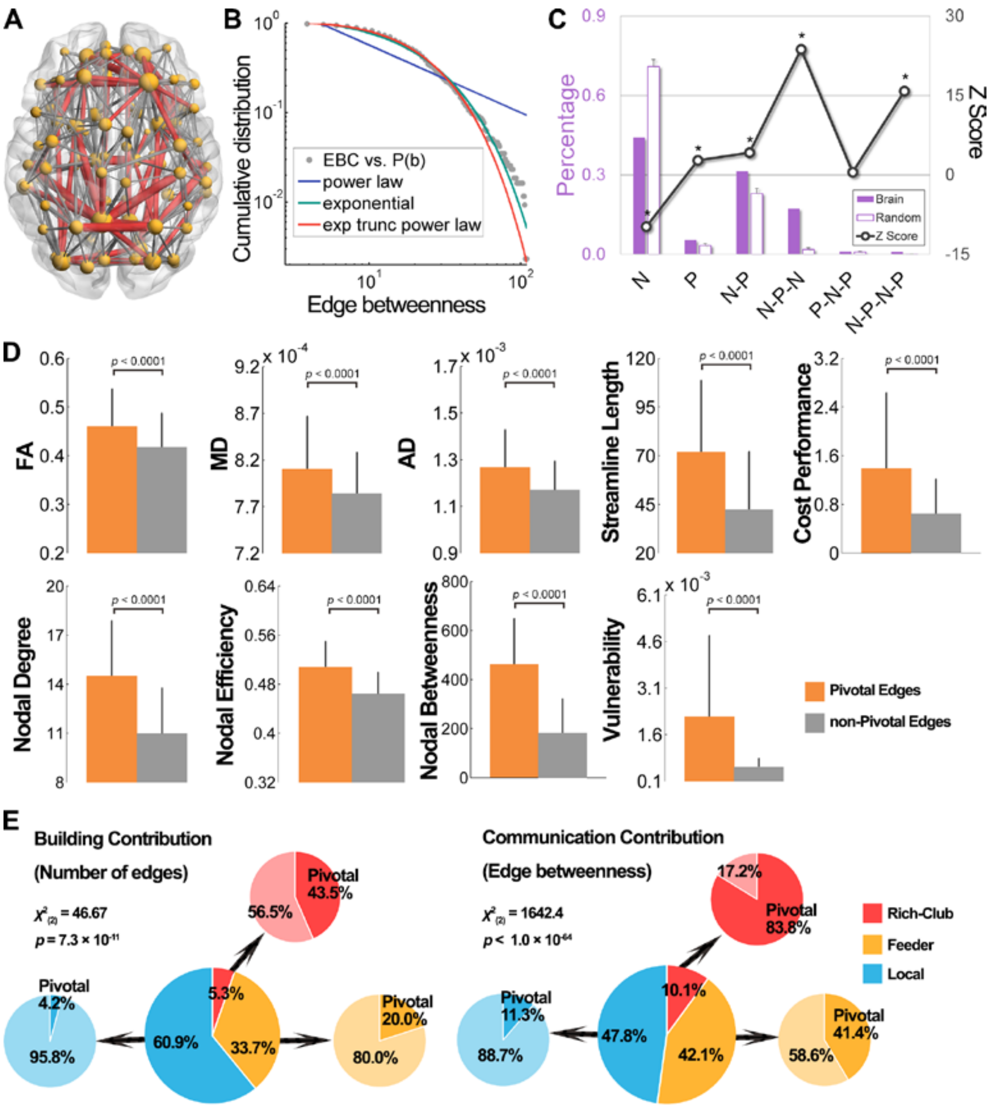

Figure S3. Validation in data of session 2. A) The spatial pattern of the pivotal edges (colored in red) of the group-level network generated from data of session 2 was highly similar with the group-level network of session 1. **B)** The edge betweenness centrality distribution of the individual WM network was best fitted by an exponentially truncated power-law form [$P(x) \sim \alpha x^{\beta} \exp(x/\gamma)$] (red, $R^2 = 0.997$) rather than power-law [$P(x) \sim \alpha x^{\beta}$] (blue, $R^2 = 0.812$) and exponential [$P(x) \sim \alpha \exp(\beta x)$] (green, $R^2 = 0.992$) models. **C)** The frequency percentage and normalized distribution of path motifs were derived by comparing the appearing frequency of each path motif to that of 1,000 equivalent random networks. The ‘non-pivotal to pivotal to non-pivotal’ (N-P-N) path motif was the most frequent path motif in the brain network (Z = 23.7). **D)** The pivotal edges showed a significantly higher level of microstructural organization (as indicated by FA, MD and AD), longer streamline length, better cost-performance, greater vulnerability and more significant contributions to all three nodal properties (nodal degree, efficiency and betweenness, respectively) than the non-pivotal ones. The error bars represent the standard deviation. **E)** The building contribution (indicated by the proportion of number) and communication contribution (indicated by the proportion of edge betweenness centrality) of the pivotal edges were significantly different ($\chi^2_{(2)} = 46.67$, $p = 7.3 \times 10^{-11}$; $\chi^2_{(2)} = 1642.4$, $p < 1.0 \times 10^{-64}$) across three categories of connections. All these results are highly consistent with the main findings on data of session 1. N, non-pivotal; P, pivotal; FA, fractional anisotropy; MD, mean diffusivity; AD, axial diffusivity.
